# Multitask Learning of Signaling and Regulatory Networks with Application to Studying Human Response to Flu

**DOI:** 10.1371/journal.pcbi.1003943

**Published:** 2014-12-18

**Authors:** Siddhartha Jain, Anthony Gitter, Ziv Bar-Joseph

**Affiliations:** 1 Computer Science Department, Carnegie Mellon University, Pittsburgh, Pennsylvania, United States of America; 2 Microsoft Research, Cambridge, Massachusetts, United States of America; 3 Department of Biological Engineering, Massachusetts Institute of Technology, Cambridge, Massachusetts, United States of America; 4 Lane Center for Computational Biology and Machine Learning Department, Carnegie Mellon University, Pittsburgh, Pennsylvania, United States of America; Princeton University, United States of America

## Abstract

Reconstructing regulatory and signaling response networks is one of the major goals of systems biology. While several successful methods have been suggested for this task, some integrating large and diverse datasets, these methods have so far been applied to reconstruct a single response network at a time, even when studying and modeling related conditions. To improve network reconstruction we developed MT-SDREM, a multi-task learning method which jointly models networks for several related conditions. In MT-SDREM, parameters are jointly constrained across the networks while still allowing for condition-specific pathways and regulation. We formulate the multi-task learning problem and discuss methods for optimizing the joint target function. We applied MT-SDREM to reconstruct dynamic human response networks for three flu strains: H1N1, H5N1 and H3N2. Our multi-task learning method was able to identify known and novel factors and genes, improving upon prior methods that model each condition independently. The MT-SDREM networks were also better at identifying proteins whose removal affects viral load indicating that joint learning can still lead to accurate, condition-specific, networks. Supporting website with MT-SDREM implementation: http://sb.cs.cmu.edu/mtsdrem

## Author Summary

To understand why some flu strains are more virulent than others, researchers attempt to profile and model the molecular human response to these strains and identify similarities and differences between the resulting models. So far, the modeling and analysis part has been done independently for each strain and the results contrasted in a post-processing step. Here we present a new method, termed MT-SDREM, that simultaneously models the response to all strains allowing us to identify both, the core response elements that are shared among the strains, and factors that are uniquely activated or repressed by individual strains. We applied this method to study the human response to three flu strains: H1N1, H3N2 and H5N1. As we show, the method was able to correctly identify several common and known factors regulating immune response to such strains and also identified unique factors for each of the strains. The models reconstructed by the simultaneous analysis method improved upon those generated by methods that model each strain response separately. Our joint models can be used to identify strain specific treatments as well as treatments that are likely to be effective against all three strains.

## Introduction

The relative ease of high-throughput data collection enables profiling a system of interest in many ways with complementary assays, at different times, and under various perturbations to compare and contrast the outcomes. The resulting computational challenge is to develop analysis strategies that maximally leverage these related experiments to improve our ability to reconstruct biologically accurate models.

Even when applied to study the same condition, different types of high-throughput data (e.g., functional genetic screens and gene expression) often times implicate largely disjoint groups of genes or proteins because each experiment highlights different facets of the biological processes and networks involved [1]. Consequently, there has been extensive research to develop techniques for integrating one or more types of *condition-specific* high-throughput data with *general purpose* physical interaction networks, such as protein-protein interactions (PPIs), to reconstruct signaling and regulatory networks [1]–[3] (see [4] for a review). These methods discern how the genes identified in complementary types of experiments relate to one another in a network context and propose new condition-specific regulators that are not directly observed to be relevant in the original data but form connections in the inferred networks.

Due to the dynamic nature of biological systems, especially those controlling stimulus response and development, it is critical to observe genome-wide changes over time [5]. As reviewed in [5], there are now computational approaches that exploit the unique structure in temporal datasets (e.g., time series gene expression) to model dynamic processes and reverse engineer regulatory networks [6], [7]. Recent algorithms integrate temporal data and PPI networks to improve signaling pathway prediction by capitalizing on the dynamic information [8], [9].

Despite advances in modeling the temporal dimension and different types of assays per condition, there has been considerably less progress made for datasets that contain multiple related perturbations or stimuli. Typically each condition is analyzed in isolation, and a post-processing comparison of the independent models is required to draw conclusions across conditions [9], [10]. Individual models of related conditions are required to appreciate the unique aspects of each, but building these models independently ignores that the observations may be generated from structurally similar networks. As an example, consider the case of host gene expression following virus infection. Although different viruses do not have identical effects on the host (hence the gene expression patterns are unique to each virus), they also commonly affect a similar core set of host proteins. These include Toll-like receptors (TLRs), which recognize a large number of RNA viruses and activate a downstream pathway that leads to common expression response [11], [12], and other elements of innate immune response pathways [13]. Similarly, in yeast several different types of stresses activate a large common set of genes (termed the environmental stress response genes [14]), and additional examples abound in other species.

When modeling such responses, one may be able to take advantage of these commonalties without sacrificing the ability to reconstruct individual models for each response. This type of machine learning is termed *Multi-task* learning [15] and usually applies to cases where one learns models for different problems that share information and/or parameters. A key advantage of such framework is the ability to utilize additional data from related conditions when reconstructing networks for a specific response. This is especially important when reconstructing biological response networks from high-throughput data because the number of parameters to fit is very large relative to the number of samples. In addition, extensive data from a well-characterized condition may be able to compensate for sparse data in a similar, less-understood condition.

Multi-task learning has been applied to other problems in the biological domain including classification [16], genome-wide association studies [17], [18], protein structure [19], and pairwise protein-protein interaction prediction [20], [21]. Multi-commodity flow [22] and iterative applications of a prize-collecting Steiner forest algorithm [23] have been used to simultaneously reconstruct related response or disease networks, but these methods do not employ multi-task learning. In addition, these previous approaches operate on static data and cannot account for the dynamic behaviors that are crucial for understanding many types of stimulus responses.

Here we present the Multi-Task Signaling and Dynamic Regulatory Events Miner (MT-SDREM), which uses multi-task learning to reconstruct response pathways and temporal regulatory networks. MT-SDREM is equipped to capitalize on the many dimensions in complex systems biology datasets by integrating different types of experimental data in each condition, explaining the time-dependent elements of a response (as observed in gene expression data), and constraining the inferred networks to be similar for related conditions or perturbations. Like its single-condition predecessor [8], MT-SDREM iterates between finding pathways that connect the upstream proteins that directly interact with an external stimulus (called source proteins) and the downstream transcription factors (TFs) that regulate the response and learning dynamic regulatory networks activated by these TFs. The learning process involves the simultaneous reconstruction of several such networks. While a different network is learned for each condition, the joint learning framework allows sharing and/or constraining parameters across the different networks which helps overcome the overfitting problem that is often an issue when reconstructing biological networks.

We demonstrate how MT-SDREM can be used to gain insights into a clinically-relevant problem: characterizing the human response to viral infection. In particular, we explore the differences between mild, seasonal strains of the influenza A virus, which are typically H1N1 or H3N2 strains [24], and lethal, pandemic strains such as the H1N1 1918 Spanish flu and highly pathogenic avian H5N1 strains. Influenza A strains are subtyped and named by their hemagglutinin (HA) and neuraminidase (NA) proteins. Although there are presently 18 known HA subtypes and 11 NA subtypes [25] only a fraction of these have have infected humans. Previous studies have characterized some of the differences between seasonal and pathogenic strains. Seasonal H1N1 and H3N2 and highly pathogenic H5N1 influenza strains infect macrophages at similar rates, but H3N2 and H5N1 causes apoptosis more rapidly than H1N1 [24]. H1N1 also lead to weaker induction of MAPK signaling pathways than the H3N2 and H5N1 strains [24]. Genomic comparisons of human and avian influenza strains identified 52 species-associated positions that could potentially enable an avian strain to cross over to humans if mutated [26]. Influenza strains also vary in the cells and tissues they infect [27], [28] with highly-virulent strains causing more widespread inflammation, including in the alveoli [27]. Highly pathogenic strains have been shown to induce a stronger inflammatory cytokine response than seasonal influenzas [28] and the host inflammatory response is often more deadly during infection than the pathogen itself [29]. However, much remains unknown about the host factors that are required for viral replication or to mount cellular defenses.

We study three strains of the influenza A virus — seasonal H1N1, seasonal H3N2, and highly pathogenic avian H5N1 — to explain how common host proteins react to the viral infection in a similar manner despite the differences in the temporal transcriptional programs that are activated. The MT-SDREM networks identified many known regulators of influenza response and also suggested putative novel regulators. Because the responses are jointly modeled using the multi-task setting, MT-SDREM is able to correctly recover TFs that are important drivers of the immune response that are missed when each viral strain is analyzed independently [10] and by previous methods for combining gene expression data across experiments. In addition, MT-SDREM networks are more predictive of host genes that are required for viral replication, a potentially clinically-relevant phenotype [29], than corresponding independent models or gene prioritization algorithms.

## Results and Discussion

MT-SDREM simultaneously infers signaling and dynamic regulatory networks for multiple related conditions. It extends the SDREM tool [8], [10] which discovers signaling pathways by orienting edges in protein interaction networks. To demonstrate the performance of such multi-task network learning we looked at data from 3 different flu viruses: H1N1, H3N2, and H5N1.

For each of these viruses we obtained time series gene expression measurements of cells infected with the virus. For H1N1 the data is from [30] and contains 10 time points. For H5N1, we obtained data from [31] with 5 time points, and the H3N2 data from [32] had 6 time points. In addition, for each of these viruses we obtained a set of sources (host proteins interacting with the virus proteins) from mass spec experiments. Data for H1N1 is from [33] and literature [30], [34] and contains 200 human proteins that were experimentally determined to interact with H1N1 proteins. Data for H3N2 is from [33] and consists of 153 host proteins and source data for H5N1 is from [33] and literature [34]–[39] and consists of 41 sources.

### MT-SDREM reconstructed networks

#### Joint signaling network

**Figure 1:**
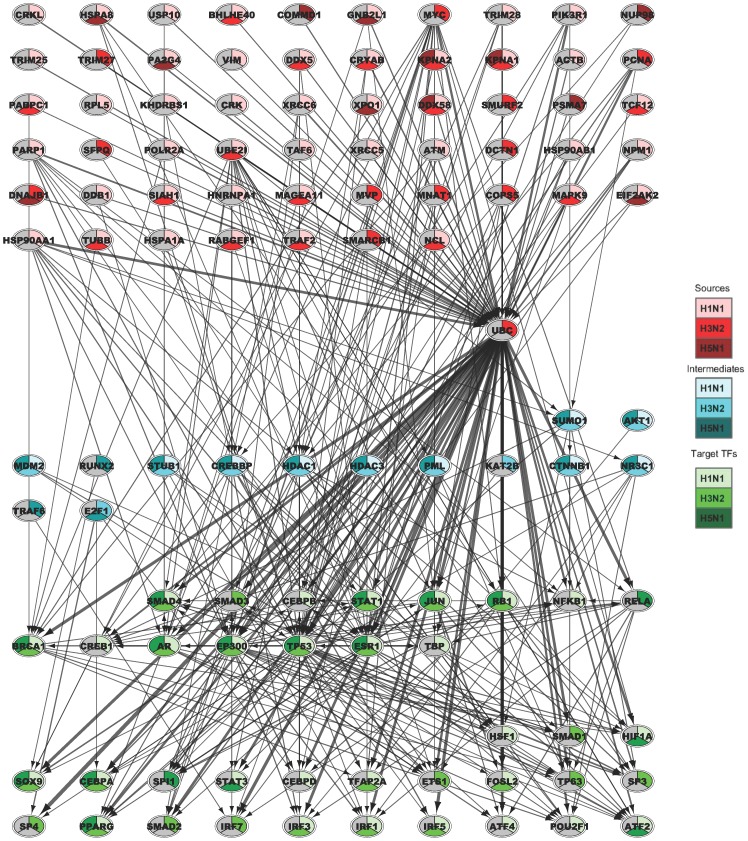
Joint signaling network inferred by MT_SDREM for the three flu viruses. Top: Sources, Middle: Signaling (intermediate) proteins, Bottom: TFs. Nodes are colored according to the role the protein is determined to play in the pathway (red - source, blue - signaling, green -TF). Each node is also denoted with the set of strains it was predicted for (color shades). For example, JUN is a TF predicted for all three strains whereas TCF12 is identified as a source for H1N1 and H3N2 but not for H5N1. See Supplementary Methods in S1 Text for details about how nodes and edges are selected from the global network for this figure.

Figure 1 presents the joint signaling network learned for the three conditions (Methods). The top layer (nodes colored in red) are sources for at least one of the conditions. The bottom layer (nodes colored green) are TFs identified in at least one of the conditions, and the middle layer (blue nodes) are signaling proteins linking the sources and TFs in the networks. We colored each node with multiple colors depending on the condition for which it was identified as a top network protein (Methods). The lightest shade for each color represent nodes from the H1N1 reconstructed network, the darkest is from the H5N1 network and the middle shade is for the H3N2 network.

While sources (red shades) are provided as inputs, all other nodes were automatically identified by MT-SDREM. Several of the proteins identified in multiple networks, both as intermediate and as TFs are well known immune response regulators. For example, we identify a pathway from UBE2I (a source for both H1N1 and H3N2) to SUMO1 (signaling protein identified for all strains). SUMO activates E1 and transfers it to conjugating enzyme E2. Then UBE2I interacts with and transfers SUMO to a target viral protein. Indeed, it has been recently shown that SUMO interacts with the key flu protein, NS1, via UBE2I [40]. In addition, TRAF6, part of the TRAF (TNF receptor associated factor) family of proteins, is identified as an important protein for H5N1. Pro-inflammatory cytokines including TNF-*α* are known to be hyper-induced in H5N1 infected macrophages [41].

We also identify several TFs as common amongst the 3 conditions. SMAD4 is present in all 3 conditions. The SMAD family of TFs is part of the TGF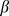 pathway which is responsible for regulating macrophage activation and proliferation of T cells [12]. STAT1 and JUN, both key immune response regulators, are also identified in all 3 conditions. We also identify NR3C1 which produces the GR protein that is known to inhibit T and B cells as well as suppressing phagocyte function [42] (this could be a viral strategy to reduce the effects of immune response). Interestingly, we identify the AKT1 gene in all 3 conditions, part of the PI3K/AKT pathway, which has recently been shown to be activated by the influenza A virus's NS1 protein [43]. We also identify the PPARG TF which has been linked to immune response by regulation of immune and inflammation related genes [44]. Other TFs belonging to the AP-1 TF complex are also identified for various conditions – ATF2 for H1N1 and H5N1, and FOSL2 for H1N1 and H3N2. NFKB1 and RELA, both part of the NF-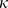
B complex are identified for H1N1 and H5N1 respectively.

#### Regulatory networks

**Figure 2:**
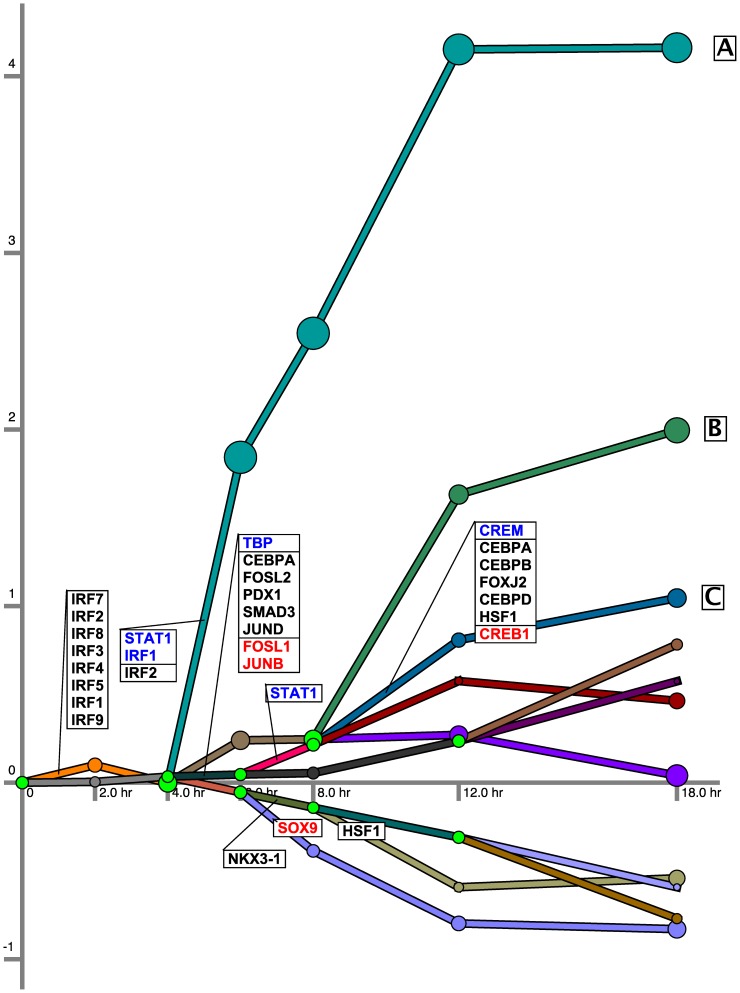
H1N1 Regulatory network. Each path represents a set of genes with a similar expression profile. Split nodes are colored green and are annotated with the TFs that are predicted to regulate genes in the paths going out of the split at the time point associated with the split. The blue TFs are up-regulated at that split time point while the red TFs are down-regulated. The black TFs are not differentially expressed at the split point. Note that several of the TFs included in this latter group are likely post-transcripitionally regulated.

In addition to the signaling parts of the networks, MT-SDREM also reconstructs dynamic regulatory networks for each of the different flu strains. We show the regulatory network inferred for H1N1 in Figure 2. For space reasons not all TFs presented in Figure 1 are shown for the model in Figure 2, though all TFs that are associated with H1N1 are used by the model. Full list of TF assignments to paths in the regulatory networks is available on the Supporting Website. Corresponding networks for H3N2 and H5N1 are presented in Supplementary Results in S1 Text. Several of the TFs identified as controlling the first splits in both the H1N1 and H3N2 networks belong to the IRF family of TFs, known to regulate interferons, which play an important role in viral immune response [12]. TFs belonging to the FOS, ATF, and JUN families appear in both the H1N1 and H5N1 networks. These TFs are part of the AP-1 TF complex (which is known to regulate gene expression in response to a variety of stimuli including cytokines, and viral infections [45]). We also identify the SMAD family of TFs to play a part in all 3 networks. The STAT family of TFs is found to play a role in all 3 conditions. This family of TFs is part of the JAK-STAT signaling pathway. This is a class of pathways responsible for activating transcription in response to extracellular signals from messengers such as interferons, interleukins, growth factors, etc. [46], [47].

In addition to analyzing the TFs identified we performed an enrichment analysis using the Gene Ontology (GO) terms associated with each path in the reconstructed regulatory networks. All p-values that we give below are after correcting for multiple hypothesis testing.

In the H1N1 regulatory network, the gene cluster corresponding to the path labeled **A** is predicted to be regulated by STAT1, part of the JAK-STAT signaling pathway, IRF1 and IRF2. This path is enriched for 'defense response to virus', 'immune response', 'type I interferon signaling pathway', and 'cytokine-mediated signaling' categories (p-value of 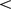
0.001 for both). We also find enrichment for similar categories in paths labeled **B**, **C** of the H1N1 network and the paths labeled **D-H** of the H3N2 network (Figure S1 in S1 Text). In addition, we also find enrichment for 'toll-like receptor signaling pathway' in path **F**, and 'T cell activation' and 'lymphocyte activation' in path **H** (p-value of 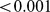). Path **D** is also predicted to be regulated by several members of the IRF family.

We find enrichment for the more general categories of 'defense response' and 'immune response' in the path labeled **I** of the H5N1 network (Figure S2 in S1 Text, p-value of 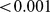). Notably, in all 3 conditions, the genes in the relevant paths are being upregulated indicating a response to all three pathogens that has shared features.

The complete list of GO categories for all the labeled paths can be found on the Supporting Website.

#### Strain specific proteins

In addition to looking for common response, we used MT-SDREM to identify strain-specific factors and proteins. These represent potential targets for individual strains and may explain why some are more virulent than others. S1 Table present the set of unique proteins identified for each strain (defined as those appearing in the top 100 proteins set for that strain, but not in the top 100 of the other two). While many of the proteins on the list are not well characterized in the three conditions making it hard to validate the results, some are known and the results agree with very recent experimental data. For example, IRF7 which was only identified by MT-SDREM for H3N2 was recently tested for H5N1 and shown to be significantly lower in H5N1 response when compared to less virulent strains [48]. Similarly, as mentioned above, the regulatory networks for H1N1 and H3N2 contain several IRFs as key regulators while the networks reconstructed for H5N1 does not pointing to a potential target for improving prognosis from this infection.

Several proteins that are only predicted for H5N1 response are known to have important roles in H5N1 infection. Knockdown of DDX39B, also known as UAP56, decreased H5N1 viral titre nearly 10 fold in infected cells [49]. MAPK8 (JNK) was strongly induced in H5N1 (and H3N2) infection, but not H1N1 infection [24]. NUP98 recruits the H5N1 protein NS2 to the nucleoli, and disrupting this interaction impedes viral propagation [38]. Mice with wild type MX1 were protected against infection by a highly lethal H5N1 strain relative to mice with defective MX1 [50]. H5N1-derived NS1 stimulates the ERK pathway, increasing cell viability and promoting infection [51]. Through interactions with viral NS1 and another host factor, IVNS1ABP (NS1-BP) can counteract this NS1-induced ERK phosphorylation [51].

### Comparison of MT-SDREM with prior work

To test the advantages of multi-task learning we compared MT-SDREM with previous methods that can be used to analyze expression and interaction data. Since we are not aware of prior methods that utilize multi-task learning in biological network reconstruction we first looked at the differences between applying MT-SDREM and applying SDREM separately to each of the three flu datasets. We have also compared MT-SDREM's results to a baseline *joint* ranking of differentially expressed (DE) genes from different experiments in a single analysis. This approach is similar to several previous studies that perform follow up analysis using such joint sets [52].

Since the 'ground truth' (complete underlying networks for each condition) is obviously unknown, we used three different types of complementary information for these comparisons. First, we examined the set of TFs identified by each of these methods and determined their relevance to the condition being studied. Next, we used the Gene Ontology (GO) to test the differences in the identified functional categories between the different analysis methods. Expression experiments and RNA interference (RNAi) screens have revealed a multitude of host pathways and processes that are involved in viral host response including MAPK signaling, apoptosis, trafficking, mRNA export, splicing, and proteolysis [30], [53], [54]. A statistical meta-analysis implicates nearly 3000 host genes [55] in these pathways. Although many processes as a whole are relevant to influenza response, not all genes participating in those processes necessarily are important. Therefore we focused our TF and GO evaluation on immune processes, which were shown to compose a critical component of the host response that kills infected cells, protects uninfected cells, combats viral components, and promotes inflammation [56] Finally, we used a set of RNAi experiments that were performed for H1N1 and H5N1 to test the ability of these different methods to identify key disease related proteins. In these experiments proteins are knocked down one at a time and the impact on viral load is measured. A protein affecting viral load is likely participating in the host response and so methods that can identify such proteins more accurately are in better agreement with the observed response. The RNAi data for H1N1 was obtained from [30], [53], [54], [57], [58] resulting in a total of 980 screen hits, 925 of which were present in our initial interaction network (which contained 16671 genes, Methods). 32 screen hits for H5N1 were obtained from [57], all of which are present in our interaction network.

### Comparison of identified TFs

In Table 1 we present the overall and pairwise overlap of the inferred TFs for the 3 conditions (extracted by same mechanism as in SDREM [8], [10]) for MT-SDREM and compare it to when SDREM is run independently on the 3 conditions (I-SDREM). Note that the pairwise intersections shown are *in addition* to the overall intersection between all of the 3 conditions.

**Table 1:** TF comparison for I-SDREM and MT-SDREM.

H1N1 & H3N2 & H5N1		H1N1 & H3N2		H3N2 & H5N1		H1N1 & H5N1	
I	MT	I	MT	I	MT	I	MT
AR	AR	IRF1	IRF1	AHR◊		EP300	ATF2 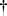
BRCA1	BRCA1	IRF3	IRF3	JUN		RELA◊	HIF1A 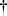
ESR1	ESR1	FOSL2	FOSL2	PPARG		TP53	STAT3 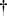
STAT1	STAT1	CEPBA	IRF5 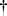	RB1			
	CEBPA 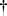	NR3C1◊	TFAP2A 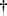	SMAD4			
	EP300 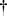	SMAD3◊		SOX9			
	JUN 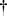						
	PPARG 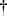						
	RB1 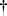						
	SMAD4 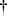						
	SOX9 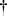						
	TP53 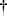						

TFs predicted to regulate two or all three response networks. Each set of conditions is divided to two columns with the first column containing TFs at the intersection of the SDREM output for the conditions and the second the MT-SDREM results for these conditions. TFs identified by MT-SDREM but not SDREM have a 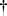 next to them and vice versa have a ◊ next to them. Note that TFs listed for the pairwise overlap are **in addition** to the ones listed for the overall overlap. Thus JUN in the I-SDREM column of H3N2 & H5N1 is not highlighted since it was identified by MT-SDREM for all three conditions.

The shared TFs identified by MT-SDREM among all 3 conditions that are missed by I-SDREM include several that are known to be immune response related. In particular, CEBPA is known to be responsible for regulating a large variety of cell functions including immune and inflammatory response [59]. MT-SDREM also identifies SMAD4 in all three conditions. SMAD family proteins are part of the TGF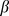 pathway as mentioned above. MT-SDREM also identifies RB1 which has been implicated in viral immune response [60], JUN which is part of the AP-1 TF complex, and PPARG an important TF regulating immune response mentioned above. In contrast, I-SDREM does not identify any TF in the intersection that MT-SDREM does not.

In addition, we also find several immune response related TFs in the pairwise overlaps for MT-SDREM that we do not see for I-SDREM. For the overlap between H1N1 and H3N2, MT-SDREM identifies IRF1/3/5 which are known to regulate interferons and thus important for immune response. For the overlap between H1N1 and H5N1, MT-SDREM finds the the STAT3 gene which is part of the JAK-STAT signaling pathway and ATF2, part of the AP-1 TF complex.

For the pairwise intersection of H1N2 and H3N2, I-SDREM identifies NR3C1 as a TF while MT-SDREM only selects it as an intermediate (signaling) protein. It also identifies another member of the SMAD family (SMAD3 whereas MT-SDREM identifies SMAD4). For H3N2 and H5N1 it identifies AHR whose activation inhibits inflammation [61] and RELA in the intersection of H1N1 and H5N1, which as part of the NF-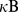 complex.

We also compared MT-SDREM to the popular TF prediction tool oPossum [62]. Our primary goal when comparing MT-SDREM with oPossum is to highlight the fact that using network information in the multi-task learning framework is useful. The input to oPossum is a list of genes identified by the experiment(s) and using this list it attempts to find overrepresented TF-binding sites. To select a common gene list from all three experiments we ranked the genes for each condition according to their differential expression and then merged the 3 rankings using the Kemeny-Young method [63]. Similar to the number of genes used by MT-SDREM we used the top 3000 in the joint ranking as input to oPossum. In Table 2 we present the comparison. Note that since we used oPossum as the tool for the comparison of MT-SDREM with other methods for integrating data from several conditions, the results shown for Table 2 are different from the intersection results of Table 1. Here, for the MT-SDREM rankings we used the *sum* of % path flow going through each gene across the 3 networks to rank TFs (Methods). The oPossum TFs are ranked according to their Z-score.

**Table 2:** TF comparison for oPossum and MT-SDREM.

oPossum	MT-SDREM
MZF1_1–4	EP300
SP1	TP53
ZNF354C	BRCA1
MZF1_5–13	JUN 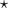
NFYA	ESR1
ZEB1 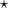	AR
MIZF	RB1 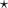
ROAZ	SMAD4 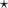
GABPA	STAT1 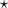
TEAD1	CEBPA 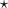
TLX1-NFIC	PPARG 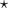
SPIB	STAT3 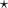
Hand1-Tcfe2a	SMAD3 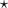
ARNT-AHR 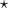	HIF1A
ELF5	RELA 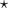
MYC-MAX	MYC
TP53	ATF2 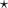
ELK1	CEBPB 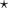
REL 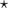	SOX9
AR	IRF1 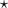

oPossum and MT-SDREM comparison. Immune response related TFs have a 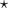 next to them. oPossum TFs are ranked according to their Z-score. MT-SDREM TFs are ranked according to the path flow measure as described in the main text and Supplementary Methods in S1 Text.

While oPossum is able to identify a few relevant TFs, for most of the TFs identified by oPossum, we could not find significant roles in immune response regulation for them. In contrast, several of the shared MT-SDREM TFs that are not identified by oPossum are known to play major roles in immune response as discussed above. These include STAT1/3, JUN/ATF2, CEBPA/B which regulate a large number of immune response genes, RB1 which has been implicated in viral immune response networks [60], PPARG, and SMAD. MT-SDREM also uniquely identifies IRF1 which plays a major role in viral immune response by regulating interferons. oPossum was able to identify only two relevant TFs that were not found by MT-SDREM. These are ZEB1 which regulates the IL2 interleukin, part of the immune response system and AHR, part of the ANTR-AHR complex. See also Tables S13–S15 in S1 Text for condition-specific comparisons using oPossum.

We also tried to compare MT-SDREM with the Inferelator method [6] but following email discussions with the authors of that method determined that such comparison is not feasible since Inferelator requires expression data for a large number of conditions while we only had time series response for three types of infections.

### GO enrichment comparisons

To compare the GO enrichment of shared genes/proteins we examined the top 500 genes in the combined MT-SDREM network (ranked using the same sum of % of path flow going through genes across the 3 networks as we did for the oPossum comparison) with the top 500 genes from the combined ranking of the differentially expressed (DE) genes from each condition (combined using the Kemeny-Young method as explained before). We used FuncAssociate [64], [65] to compute standard GO enrichment for the genes. We found **3** categories, only 2 of which were immune response related for which the p-value for DE genes was 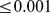 but which were not present in the MT-SDREM list or if present, their p-value was 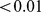. The categories are listed in Table 4. However, for the vice versa comparison, we found a large number of categories for which the MT-SDREM p-value was 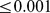 but which were either not enriched for in the DE genes list (most common outcome) or if present, their p-value was 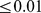. A subset of the immune response related categories are listed in Table 3. Note that we find significant enrichment for a very varied set of immune response processes including T cell activation, cytokine production, activation of immune response, etc. as well as categories related to viral genome expression and positive regulation of viral process. The DE genes list is only enriched for negative regulation of viral process and viral genome replication. The complete set of the categories is in S45 Table.

**Table 3:** GO categories enriched in MT-SDREM that are not enriched as significantly in Differentially Expressed (DE) genes.

GO Category	MT-SDREM p-value 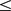	DE genes p-value	GO Category Description
GO:0002218	0.001	NA	activation of innate immune response
GO:0002684	0.001	NA	positive regulation of immune system process
GO:0002429	0.001	NA	immune response-activating cell surface receptor signaling pathway
GO:0046328	0.001	NA	regulation of JNK cascade
GO:0001816	0.001	NA	cytokine production
GO:0001959	0.001	NA	regulation of cytokine-mediated signaling pathway
GO:0042113	0.001	NA	B cell activation
GO:0042110	0.001	NA	T cell activation
GO:0043923	0.001	NA	positive regulation by host of viral transcription
GO:0019080	0.001	NA	viral genome expression
GO:0048524	0.001	NA	positive regulation of viral process
GO:0007259	0.001	NA	JAK-STAT cascade
GO:0002573	0.001	NA	myeloid leukocyte differentiation

GO comparison between the Differentially Expressed gene list and MT-SDREM gene list for top 500 genes. The enrichment was performed using the FuncAssociate tool [64]. Only categories with MT-SDREM adjusted p-value of 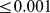 and DE genes p-value of 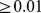 are presented. If a p-value for DE genes is NA, that means that that category was not enriched for in the DE genes list. Only **select** immune response related categories are presented. The full list of the 114 immune-related categories is available in S45 Table on the Supporting Website.

**Table 4:** GO categories enriched in DE genes that are not enriched as significantly in MT-SDREM.

GO Category	DE p-value 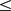	MT-SDREM p-value	GO Category Description
GO:0045071	0.001	NA	negative regulation of viral genome replication
GO:0048525	0.001	0.019	negative regulation of viral process

GO comparison between the joint DE gene list and the joint MT-SDREM for the top 500 genes. The enrichment was performed using the FuncAssociate tool [64]. Only categories with DE genes adjusted p-value of 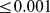 and MT-SDREM genes p-value of 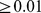 are presented. If a p-value for MT-SDREM is NA, that means that that category was not enriched for in the MT-SDREM list. **All** immune response related categories are presented.

To further compare methods that are based on joint expression analysis to those that are based on joint network learning we looked at the GO enrichment for the top 50 TFs identified by MT-SDREM and oPossum. The top 50 TFs for MT-SDREM are ranked using the joint ranking based on path flow for the 3 conditions as done for the GO comparison above. We used the TF Z-score provided by oPossum to rank TFs for oPossum. We again used FuncAssociate [64], [65] to compute standard GO enrichment for the TFs. We obtained only one immune-response related category (interleukin related) for which the p-value for the oPossum TF set was 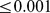 while being 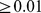 for MT-SDREM (presented in Table 6). However we obtained 270 categories in total for which the MT-SDREM p-value was 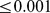 but the p-value for oPossum was 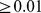, a large number of which were immune response related. Due to space constraints, only a subset of these are presented in Table 5. These include 'postive regulation of innate immune response', 'viral process', and 'cytokine-mediated signaling pathway'. The complete list of categories is in S46 Table. See also Tables S14–S25 in S1 Text for several additional comparisons of MT-SDREM and other methods using GO enrichment data.

**Table 5:** GO categories enriched in MT-SDREM TFs that are not enriched as significantly in oPossum TFs.

GO Category	MT-SDREM p-value 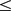	oPossum p-value	GO Category Description
GO:0009607	0.001	NA	response to biotic stimulus
GO:0045089	0.001	0.02	positive regulation of innate immune response
GO:0071357	0.001	NA	cellular response to type I interferon
GO:0019048	0.001	NA	modulation by virus of host morphology or physiology
GO:0016032	0.001	NA	viral process
GO:0019221	0.001	NA	cytokine-mediated signaling pathway
GO:0032481	0.001	NA	positive regulation of type I interferon production
GO:0046332	0.001	NA	SMAD binding

GO comparison between the joint oPossum TF list and the joint MT-SDREM TF list for the top 50 TFs. The comparison was performed using the FuncAssociate tool [64]. A subset of the categories for which the MT-SDREM list p-value is 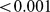 and that of the oPossum list is 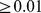 or which are not enriched for the oPossum list (represented by NA as the p-value) and which are immune response related are presented. Note that only a subset of the 40 immune-related categories are presented. The rest of the categories are available in S46 Table on the Supporting Website.

**Table 6:** GO categories enriched in oPossum TFs that are not enriched as significantly in MT-SDREM TFs.

GO Category	oPossum p-value 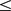	MT-SDREM p-value	GO Category Description
GO:0045084	0.001	NA	positive regulation of interleukin-12 biosynthetic process

GO comparison between the joint oPossum TF list and the joint MT-SDREM TF list for the top 50 TFs. The comparison was performed using the FuncAssociate tool [64]. Categories for which the oPossum list p-value is 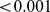 and that of the MT-SDREM list is 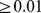 or which are not enriched for the MT-SDREM list (represented by NA as the p-value) and which are immune response related are presented. Note that **all** immune related categories are presented.

### RNAi screen hits

**Figure 3:**
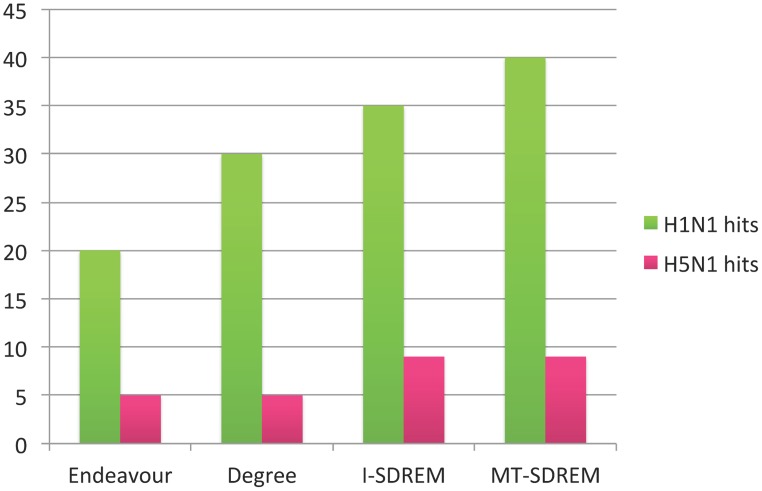
Screen hits overlap for top 100 ranked genes for both H1N1 and H5N1. 925 H1N1 and 32 H5N1 screen hit proteins were present in our network.

Using the screen hit data for H1N1 and H5N1 we compared the performance of MT-SDREM, I-SDREM and Endeavour [66], [67]. Endeavour is a gene prioritization algorithm which uses a set of seed genes (the sources) to rank genes based on several types of evidence including gene expression, interaction networks derived from various sources, text mining, sequence similarity, and functional annotations. It combines the individual rankings to create a global ranking for all genes. For the MT-SDREM and I-SDREM results we ranked proteins based on the total number of paths weighted by their score going through them. See Supplementary Methods in S1 Text for details. For Endeavour, we configured it to use only BioGRID and HPRD as data sources as those are the only sources we use to construct our PPI network. The expression data is not used by Endeavour. We gave the source proteins as the seed genes to Endeavour. We further compared these three methods with a baseline method that is condition-independent: ranking nodes by their weighted degree in the PPI network. The results are presented in Figure 3. For H1N1, the top 100 genes in the Endeavour ranking include only 20 screen hits (p-value is 4.9E-7). For I-SDREM the number increases to 35 (p-value 2.0E-19) whereas MT-SDREM obtains the highest number of protein in the overlap 39 (p-value 1.7E-23). The baseline comparison where we rank by degree has an overlap of 30 genes (p-value 9.4E-15). For H5N1, the top 100 genes for Endeavour and for ranking by degree include only 5 screen hits (p-value 1.2E-6) whereas both I-SDREM and MT-SDREM have an overlap of 9 screen hits (p-value 1.7E-13). See also S1 Text for comparison of RNAi screen hits using GSEA.

We also compared MT-SDREM, I-SDREM with GeneMania [68], [69] and concluded that MT-SDREM greatly improves upon the GeneMania results. See Supplementary Results in S1 Text for details.

### Conclusions and future work

We developed MT-SDREM a multi-task learning framework that simultaneously reconstructs signaling and dynamic regulatory networks across related conditions. Given the small number of condition-specific samples that are often available (i.e. time series expression data and host-pathogen interaction data) sharing parameters across related conditions allows the reconstruction of more accurate networks while still retaining the ability to explain condition-specific signaling and regulation.

We applied MT-SDREM to reconstruct networks for 3 related influenza A virus infections – H1N1, H3N2, and H5N1. The resulting signaling and regulatory networks were able to identify several known and novel regulators of immune and viral response. Many of these were shared between all condition including PPARG, FOS, ATF, and JUN. Similarly, we identify key signaling proteins, some shared by all conditions while others are unique to one or two of the conditions. Specifically, we identified the signaling protein SUMO1 as part of pathway from UBE2I for all 3 conditions. This agrees with recent findings that UBE2I interacts with SUMO1 to degrade influenza A's virus, NS1 which is present in all three strains [70]. We also identified the AKT1 gene, part of the PI3K/AKT pathway that is activated by NS1 in all conditions.

MT-SDREM is the first method to jointly reconstruct such dynamic networks. Comparing MT-SDREM with methods that have been suggested to integrate gene expression data or with methods reconstruct such networks independently for each condition highlighted the advantages of multi-task network learning. MT-SDREM outperformed previous methods in identifying a set of TFs controlling immune response, a set of functionally relevant proteins and a set of proteins whose knockdown affects viral loads.

While MT-SDREM can successfully utilize experiments from similar conditions to reconstruct signaling and regulatory networks, there are still issues we would like to improve in future work. One direction we intend to explore is extending MT-SDREM to allow time based (as opposed to global) sharing of TFs across conditions so that splits representing the same time will be more likely to share TFs compared to other splits. We would also like to improve on the models by using additional types of data, including epigenetic data which can help improve the priors for TF binding at specific time points by making them a function of the epigenetic code.

## Materials and Methods

MT-SDREM simultaneously investigates and infers regulatory networks and signaling pathways for several biologically related conditions. For this, it uses both condition-specific gene expression and interaction data and general interaction data. We first discuss the input data that the method utilizes and then present the modeling and learning frameworks.

### Input Data

We use 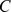 to denote the set of conditions that are jointly modeled by MT-SDREM. Below we list the datasets used by MT-SDREM.

1. *Condition-specific: Time series gene expression data* for each of the conditions that are modeled by MT-SDREM.
2. *Condition-specific: Sources* 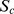 - the set of sources or host proteins which are known experimentally to interact with the pathogen/treatment applied when studying condition 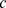.
3. *Condition-specific (optional): Screen hits* A list of proteins for each condition whose removal is known to phenotypically impact the response of the cells in that condition.
4. *General and/or condition-specific: TF-gene binding data*: A list of potential TF-gene interactions with an optional probabilistic prior/likelihood for the interaction. If data is available for the specific condition/cell type being studied these can be used, otherwise general data can be used as well. We denote by 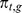 the interaction prior for TF 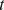 binding with gene 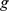.
5. *General: Protein interaction network*: A list of protein-protein interactions which may be directed or undirected. The method can also use information regarding the confidence in each interaction. We denote such confidence in edge 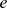 by 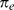 and by 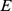 the set of all edges.

#### Protein-protein and protein-DNA interactions

We obtained a list of protein-protein interactions from BIOGRID [71] and HPRD [72]. We also use Post-translational Modification Annotations from the HPRD dataset. General Protein-DNA interactions are from [73] were processed as described in [74]. The top 100 threshold was used for both the interaction network and when analyzing the temporal expression data. As the TF binding predictions are not cell type-specific and as the H1N1 data was aggregated from multiple cell types, we assigned these predictions a relatively low score of 0.3 in the interaction network.

For the PPI network, a probability for every PPI edge was obtained by combining the various types of experimental evidence (Affinity-capture, Yeast 2-Hybrid). See Supplemental Methods in S1 Text for details. The PPI network we construct has 16,671 nodes and 228,159 edges. For 58,322 of these edges we have direction information in the database (most of those edges are TF-gene binding interactions and for the rest, the direction information primarily comes from phosphorylation studies).

#### Condition-specific data

We obtained time series gene expression data for each of the 3 viruses under consideration - H1N1 [30], H3N2 [32], and H5N1 [31] with 10, 6, and 5 time points respectively. The expression data was generated using whole genome microarray. To reduce the level of noise in the gene expression data, for every condition, we only used the top 3000 most differentially expressed genes in the time series dataset for that condition as input to MT-SDREM (see Supplementary Methods in S1 Text for details on how the genes were selected).

We collected the sources (human proteins experimentally determined to interact with the 3 viruses' proteins) from VirHostNet [33]. There are 200 sources for H1N1, 153 for H3N2, and 41 for H5N1. In addition, we included TLR3, TLR7, TLR8, RIG-I and NLRP3 [75]–[77] – proteins that either detect influenza viral RNA or influenza infection via other means – as sources for H1N1 and H5N1.

### SDREM

MT-SDREM extends the Signaling and Dynamic Regulatory Events Miner (SDREM) which has so far only been applied to reconstruct response networks for a single condition at a time [8]. Prior to discussing the multi-task learning procedures we first briefly discuss the SDREM method. SDREM is an iterative procedure that combines regulatory and signaling network reconstruction to model response pathways. For the regulatory part, SDREM uses time series gene expression data with protein-DNA interaction data to identify bifurcation events in a time series (places where the expression of previously co-expressed set of genes diverges – see Figure 2), and the transcription factors (TFs) controlling these split events. While some TFs are transcriptionally activated, others are only activated post-translationally via signaling networks. To explain these TFs, the second part of SDREM links sources (host proteins that directly interact with the virus/treatment) to the TFs determined to regulate the regulatory network. This part of SDREM uses protein-protein interaction (PPI) and protein modification data to infer such pathways – while imposing the constraint that the *direction* of PPI in the inferred pathways is consistent. These two parts (regulatory and signaling reconstruction) iterate a fixed number of times until the final network is obtained. See [8] for complete details.

#### Application of multi-task learning to the inference of signaling and regulatory networks

As mentioned above, we can run SDREM individually on the expression data for different infections to infer regulatory and signaling cascades for each of these conditions. However, several shared attributes can be jointly learned for these conditions and given the scarcity of data compared to the number of variables (very few time points for each expression experiment with thousands of genes in each model) such an approach can improve the accuracy of the reconstructed networks for each condition. Specifically, the direction of (the originally undirected) PPIs is likely to be similar for all conditions since several pathways are likely used by multiple conditions. Similarly, TFs that are active in response to one virus are more likely to be active in response to other viruses as well. MT-SDREM defines an optimization function that captures these expected similarities while still allowing for a condition-specific response component.

#### The multi-task learning objective function

The objective function commonly used for multi-task learning combines two related goals: First, similar to standard machine learning applications (for example, classification) it tries to minimize the loss (i.e. error) for each task while at the same time regularizing the parameters used by each task to avoid overfitting. Second, it further regularizes the parameters *across tasks* so that the final parameters are similar. A typical objective function is the following [78]

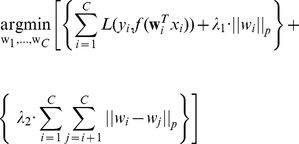
where 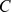 is the number of tasks, 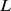 is the loss function, 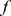 is a function of the dot product of the task-specific weight vector and the data for the task, and 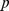 is the 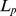 norm for the regularization. The part in the left curly braces, 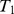 is the *task-specific* part of the objective function while the part in the right curly braces, 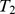 is the regularization *across* tasks.

#### Multi-task objective for MT-SDREM

In MT-SDREM, the loss minimizing part, 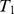, is achieved by the regulatory network learning procedure which learns parameters for a IOHMM that uses a logistic regression classifier to compute transition probabilities (Supplementary Methods in S1 Text). The logistic regression classifier is regularized using Lasso to reduce the number of active TFs inferred for each split. Thus in terms of the multi-task objective, 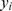 corresponds to the prediction regarding a gene trajectory at any split and 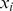 is the *TF-gene binding information*. 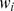 is the set of logistic regression weights learned for each split. Note that the TF-gene binding information 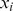 is *not* specific to each split but is the same for the entire times series.

In addition to expression data, we use signaling network information to infer TFs that are reachable from the infection sources. Such TFs are more likely to explain how the infecting agents affects gene expression and so their weights are increased in our framework. To find such TFs we need to orient the undirected edges and determine a weight for the paths leading to these TFs from sources. These two procedures (edge orientation and TF re-weighting) are shared across tasks and both affect the TF priors used by the logistic regression function. Thus for MT-SDREM, the objective function is:
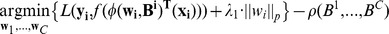
where 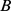 is the weight matrix learned for TFs for all tasks in the signaling network and 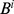 are the weights determined for task 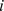. 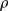 is the similarity function used to constrain parameters across tasks which is described below (hence the negative sign in front of it as we are minimizing the objective but we want to maximize the similarity).

An important difference between the standard multi-task learning framework and our method is that while we regularize the within task parameters (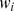
's), the between task parameters (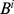
's) are not explicitly regularized. The reason is that the 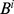
s are already constrained by the input protein interaction network and so are inherently bounded. See Supplementary Methods in S1 Text for details on the specific terms used in the multi-task learning objective.

Given 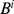, the above equation can be optimized by fitting parameters to the IOHMM and logistic regression function as was previously done in [79]. See Supplement Methods in S1 Text for details.

#### Between task regularization

Next we discuss how we use the signaling network to determine the values for , the TF weights used to reconstruct the regulatory networks. While the main goal of the regulatory network reconstruction method is to explain the temporal gene expression trajectories using the dynamic activation of TFs, the main objective when reconstructing the signaling network is to explain how these TFs are activated by the infecting viruses. For this, we attempt to link sources (protein interacting with the virus) and targets (TFs controlling virus-specific expression response) using paths in the network. The orientation is determined by specifying edge directionality to optimize the following equation:
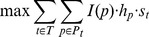
where 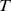 is the list of TFs predicted to regulate the time series for a specific condition, 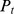 is the set of paths that start from a source of this condition and end in TF 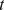, 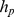 is the weight of the path which is defined as the multiplication of the probabilities of the edges in the path, and 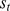 is the score of the TF 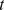 obtained from the regulatory network reconstruction. 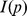 is an indicator function indicating whether path 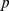 is satisfied or not (a path is satisfied if all the edges in the path are oriented in a direction that links the source to the target) and thus optimizing the above equations requires the assignment of directionality to the PPI edges (see [8], [10] for details). Note that a Breadth First Search or a Depth First Search are not enough to solve this since we assume PPI edges may be undirected. Thus, certain paths can contradict each other in terms of the specific edge direction making this a non trivial optimization problem (in fact, it is NP complete – see [79] for details and algorithm for solving this problem).

If we have multiple conditions we can simply run this function independently for each of them leading to the following set of optimization problems:
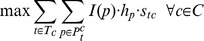



Here 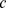 goes over each of the conditions and the function is optimized independently for that condition. However, such independent optimization may lead to contradictory directionality assignments. In addition, it does not utilize shared properties between the conditions. Instead, we would like to -

1. Constrain the model to use shared parameters – thus the direction of the edges in the signaling networks is constrained to be the same in all models.
2. Favor pathways which end in TFs that are used in more than one condition.

To achieve the first goal above we attempt to maximize the objectives for each condition using a shared, directed, network. For this we modify the search procedure by assigning edge direction to maximize the sum of the objectives across all networks. See Supplementary methods in S1 Text for details.

The second requirement is more involved since it requires us to change node scores based on TF usage across the conditions. To obtain more shared TFs we add an additional term to the objective function. We introduce a new, global, parameter, 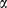 which is used to increase the weight assigned to shared TFs. See Supplementary methods in S1 Text for details. Also see Table S7 in S1 Text for discussion on the impact of different values of 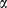 on the performance of MT-SDREM.

### Ranking proteins in reconstructed networks

Following the multi-task learning procedure we arrive at directed, weighted networks for each of the conditions being studied. To further select the key proteins from each of these networks we rank the proteins based on the "path flow" going through a node. The path flow 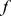 through a node 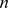 is defined as follows –
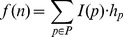
where 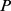 is the set of paths containing node 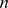.

To combine the rankings from each condition into a single ranking, we compute the total flow through all the nodes
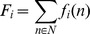
where 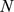 is the set of genes and 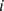 is the condition and then we computed the % flow 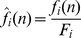 through a node. To get the combined score for a gene across conditions, we sum up the condition-specific % flows to get 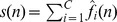 where 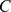 is the number of conditions. Then we rank the genes in descending order of the final score 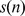.

## Supporting Information

S35 Table Extension of Table S8 in S1 Text containing all the immune related GO categories for MT-SDREM not present or with p-value ≥0.01 Tensor clustering for the complete network.
(XLSX)

S36 Table Extension of Table S9 in S1 Text containing all the immune related GO categories for MT-SDREM not present in or with p-value ≥0.01 Tensor clustering for the top 20,000 edges in the network.
(XLSX)

S37 Table Extension of Table S11 in S1 Text containing all the immune related GO categories for MT-SDREM not present in or with p-value ≥0.01 in Tensor clustering for the top 5,000 edges in the network.
(XLSX)

S38 Table Extension of Table S16 in S1 Text containing all immune-response related categories for MT-SDREM not present or with p-value ≥0.01 in DE genes for top 500 genes for H1N1.
(XLSX)

S39 Table Extension of Table S18 in S1 Text containing all immune-response related categories for MT-SDREM not present or with p-value ≥0.01 in DE genes for top 500 genes for H3N2.
(XLSX)

S40 Table Extension of Table S20 in S1 Text containing all immune-response related categories for MT-SDREM not present or with p-value ≥0.01 in DE genes for top 500 genes for H5N1.
(XLSX)

S41 Table Extension of Table S21 in S1 Text containing all immune-response related categories for MT-SDREM not present or with p-value ≥0.01 in DE genes for top 1000 genes for the joint list.
(XLSX)

S42 Table Extension of Table S23 in S1 Text containing all immune-response related categories for MT-SDREM not present or with p-value ≥0.01 in DE genes for top 1000 genes for H1N1.
(XLSX)

S43 Table Extension of Table S25 in S1 Text containing all immune-response related categories for MT-SDREM not present or with p-value ≥0.01 in DE genes for top 1000 genes for H3N2.
(XLSX)

S44 Table Extension of Table S27 in S1 Text containing all immune-response related categories for MT-SDREM not present or with p-value ≥0.01 in DE genes for top 1000 genes for H5N1.
(XLSX)

S45 Table Extension of Table 3 containing all immune-response related categories for MT-SDREM not present or with p-value ≥0.01 in DE genes for top 500 genes for the joint list.
(XLSX)

S46 Table Extension of Table 5 containing all immune-response related categories for MT-SDREM not present or with p-value ≥0.01 in oPossoum TFs for top 50 TFs based on the joint gene ranking list.
(XLSX)

S1 Text Supporting Methods, Supporting Results and Figures.
(PDF)
