# Supplementary material for: Multitask Learning of Signaling and Regulatory Networks with Application to Studying Human Response to Flu
Source: PLoS Comput Biol. 2014 Dec 18;10(12):e1003943. doi: 10.1371/journal.pcbi.1003943 (PMC4270428; doi:10.1371/journal.pcbi.1003943)
Supplement: S1 Text — Supporting Methods, Supporting Results and Figures. (PDF) [file pcbi.1003943.s013.pdf]

Supplement for Multitask learning of Signaling and Regulatory networks with Application to Studying Human Response to Flu

Siddhartha Jain[1], Anthony Gitter[2,3], Ziv Bar-Joseph[4]
1 Computer Science Department, Carnegie Mellon University, Pittsburgh, Pennsylvania, United States
2 Microsoft Research, Cambridge, Massachusetts, United States
3 Department of Biological Engineering, Massachusetts Institute of Technology, Cambridge, Massachusetts, United States
4 Lane Center for Computational Biology and Machine Learning Department, Carnegie Mellon University, Pittsburgh, Pennsylvania, United States
∗ E-mail: zivbj@cs.cmu.edu

Contents

Supplementary Methods **1**
Optimizing the MT-SDREM objective 1
Detailed description of the algorithm 2
Learning parameters for the multi-task objective 3
Constructing the joint signaling network 3
Kemeny-Young Method 3

Supplementary Results **4**
Regulatory networks for H3N2 and H5N1 4
Strain-specific protein list 6
RNAi screen hits 7
GSEA comparison 8
Stability of results with respect to α 9
Comparison to Tensor Clustering 11
Further comparison to oPossum 13
Further GO comparison to Differentially Expressed (DE) genes 16
Strain-specific statistics 22
GO comparison with I-SDREM 22

Supplementary Methods

Optimizing the MT-SDREM objective

Using α we maximize the following objective:

$$\rho(B^1, ..., B^K) \propto \sum_{c \in C} \sum_{t \in T_c} \sum_{p \in P_t^c} n_t^\alpha \cdot I(p) \cdot h_p \cdot s_{tc}$$

Here n_t is the number of conditions the TF t is predicted to regulate, where T_c is the list of TFs predicted to regulate the time series for condition c, P_t^c is the set of paths that start from a source of condition c and end in TF t, h_p is the weight of the path which is defined as the multiplication of the probabilities of the edges in the path, s_{tc} is the score of the TF t for condition c, and $I(p)$ is an indicator function indicating whether path p is satisfied or not (a path is satisfied if all the edges in the path are oriented in a direction that links the source to the target).

For $\alpha \geq 1$ the objective above would prefer selecting joint TFs to equally explanatory TFs that are not shared. Thus α represents a trade-off between fitting individual networks (specifically, $\alpha = 1$ means that we are back to our condition independent network learning) and learning a single joint network (very high values of α will lead to the selection of the same TFs for *all* networks). Note that the n_t^α factor implements the ρ function (for regularizing between tasks). The procedure to select an appropriate value for α is described later.

We use a greedy algorithm to optimize the objective. We randomly select a direction for every edge that has conflicting direction, i.e. it is present in opposite directions in two different pathways. We then do a local search to arrive at a local minimum. We flip the directions of the conflicting edges, always choosing the flip that increases our objective by the highest amount until we can't find any flip that would still improve the objective. This approach is similar to SDREM's approach which has been shown to work well, both on real and on simulated data [1].

After we optimize the above objective, we obtain a single oriented network for each condition. We then use that network to obtain new priors for TFs for DREM. First we compute the weights for the TF for each condition using the equation

$$w_t^c = \sum_{p \in P_t^c} I(p) \cdot h_p \cdot s_t$$

where t is the TF and P_t^c is the set of selected paths for condition c that end in TF t. To normalize these scores, we further run the above orientation procedure L number of times, each time with an *additional* set of randomly selected TFs which are *not* predicted to regulate *any* of the conditions. We use the random score to adjust the score for the predicted TF [1].

Detailed description of the algorithm

We constructed a probability for each protein-protein interaction (ppi) using the formula $1 - \Pi_{i=1}^{n}(1 - p_i)$ where p_i is our confidence that the ith experimental evidence for the ppi is a true positive. The network so constructed has in the HGNC symbol naming scheme, 228,159 edges and 16,671 proteins with maximum degree 9,368 and average degree 27.372.

1. First, we pre-process the data. Let $S = \cup_{c \in C} S_c$ where S_c is the set of sources for condition c. We exhaustively search for all simple (non-cylic) paths from all sources in S to all TFs in our protein interaction network. The weight of a path is defined as the multiplication of the probabilities of the edges in the path (actual calculation done by summation in log space). We select and keep the top k paths by weight. Denote this set of paths P and let the weight of path p be h_p.

2. We run DREM (Dynamic Regulatory Events Miner) on the time series data for every condition individually with default prior $\pi_{tg} = 0.5$ for all TF-gene interactions. DREM is based on an input output Hidden Markov model and annotates the various time points of the time series with TFs that are supposed to be regulating the genes at those time points.

3. We extract a list of TFs from the results output by DREM and assign them a score in the same manner as done in SDREM. Let T_c be the set of TFs for condition c and let s_{tc} be the score of TF t in condition c. Let n_t be the number of conditions TF t is present in. Let $T = \cup_c T_c$ and T_{all} be the set of all possible TFs.
   In addition, the score for a TF is increased by multiplication with the n_t^α factor discussed in the previous section. As mentioned before, this factor is how we implement the ρ function of the objective.

4. We create the TF sets $T^i = T \cup T_r$ where T_r^i is a randomly selected set of TFs from $T_{all} \backslash T$ such that $|T_r| = |T|$. We create L such sets. In addition we also create the set $T_r^{L+1} = \emptyset$ and thus $T^{L+1} = T$.

5. For every TF $t \in T^i, 1 \leq i \leq L+1$, we then compute $f_{ti} = \sum_c \sum_p h_p \cdot s_{tc}$ where the path p ends in TF t and p has edges that are of the same orientation as those reached in optimization problem i.

6. We then create a list M consisting of all the f_{ti} so computed and sort that list according to the f_{ti}.

7. Then for a TF $t \in T$, if $f_{t,L+1}$ is in the 80th percentile of list M, we increase it's prior via the formula $\pi_{tg}^{new} = (\pi_{tg} + 1)/2$ for all genes g. In addition, if $f_{t,L+1}$ is greater than the node threshold parameter, the prior is also increased similarly.
   If neither of the two conditions hold, we decrease the prior via the formula $\pi_{tg}^{new} = \max(0.01, \pi_{tg}/2)$

8. We run steps 2-7 for 10 iterations which is the default number of iterations of SDREM.

Learning parameters for the multi-task objective

For handling parameters for the SDREM component, we refer the reader to [1]. We use the default provided parameters for SDREM inside of MT-SDREM. MT-SDREM adds the α parameter to the set of parameters. α encodes our prior on how much the given conditions are related. One way to choose α is to perform cross validation on say the number of RNAi hits one obtains in the top k ranked genes. Another, which is what we use here, is to look for an α which is in a stable region – i.e. perturbing it would not change the results in terms of the TFs that we extract. We found that $\alpha = 6$ achieves such stability and we thus use that for all our experiments. Results for other α close to 6 are provided in the Supplementary Results.

Constructing the joint signaling network

To construct the joint signaling network in Figure 1 of the main text, we took the top 200 source, intermediate, and target (TFs predicted to regulated the condition's gene expression) proteins from each of the 3 conditions. We then looked at the set of paths from a source protein to an intermediate protein to a target protein and computing the condition-specific path flow for each protein (see Materials and Methods for details on how to compute it given a set of paths). We also computed the path flow for each edge between the selected proteins. Edge path flow was computed in a similar manner to node path flow by summing path scores for all paths containing that edge. We then only selected nodes which had a flow of at least 1000 and edges which had a flow of at least 200.

Kemeny-Young Method

The Kemeny-Young method is used to merge several different ranked lists $r_1, \ldots, r_n$ into a single ranked list R. R is constructed so as to minimize the total number of conflicts with the lists $r_1, \ldots, r_n$. For example, if r_i ranks gene A above gene B, but R ranks gene B above gene A, that counts as 1 conflict. The sum of such conflicts between R and $r_i, 1 \leq i \leq n$ is what is minimized by the Kemeny-Young method.

Supplementary Results

Regulatory networks for H3N2 and H5N1

The regulatory networks for H3N2 and H5N1 are presented in this section. The discussion of the figures is in the Results section of the main text.

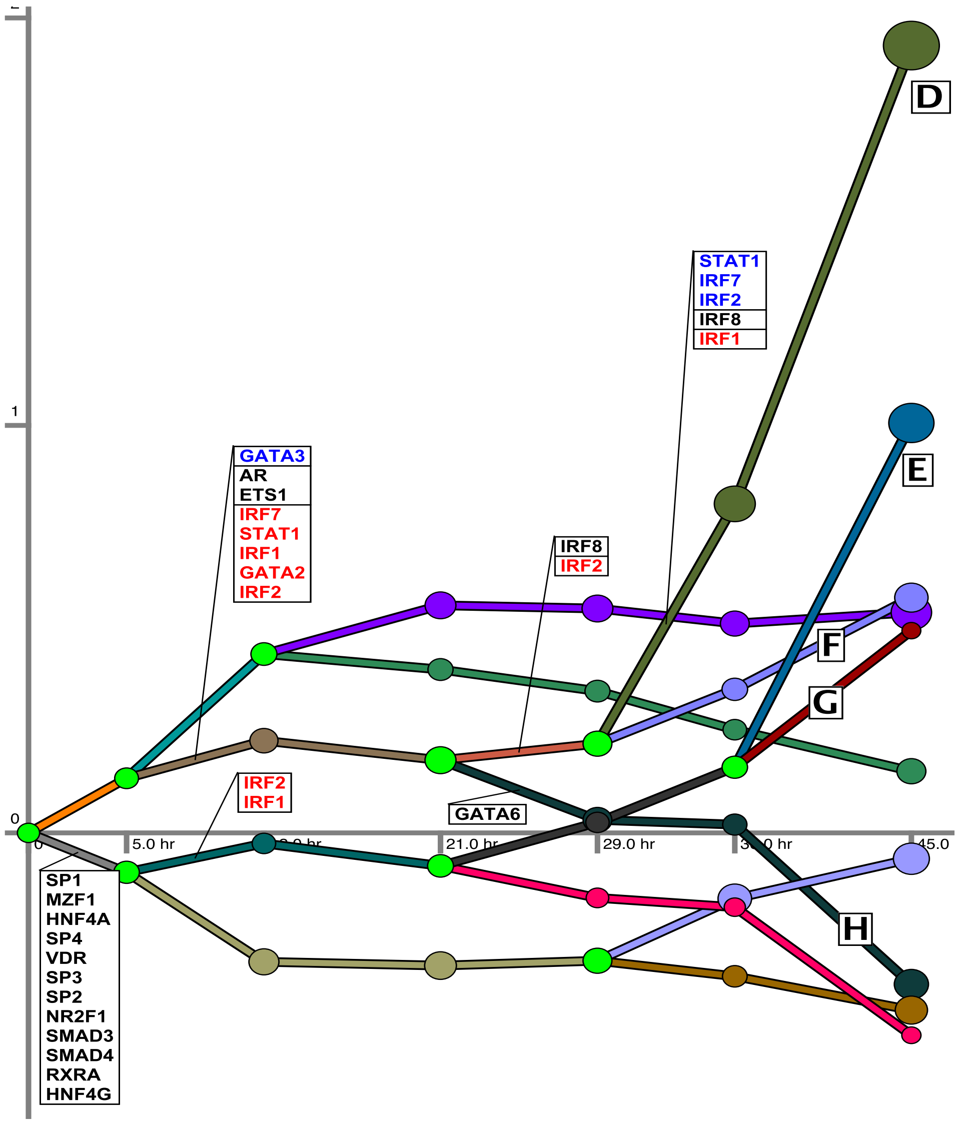

Figure S1. The H3N2 regulatory network is presented. The paths represent the different gene sets which are coexpressed. The TF lists are the TFs predicted to regulate the path that they are connected to.

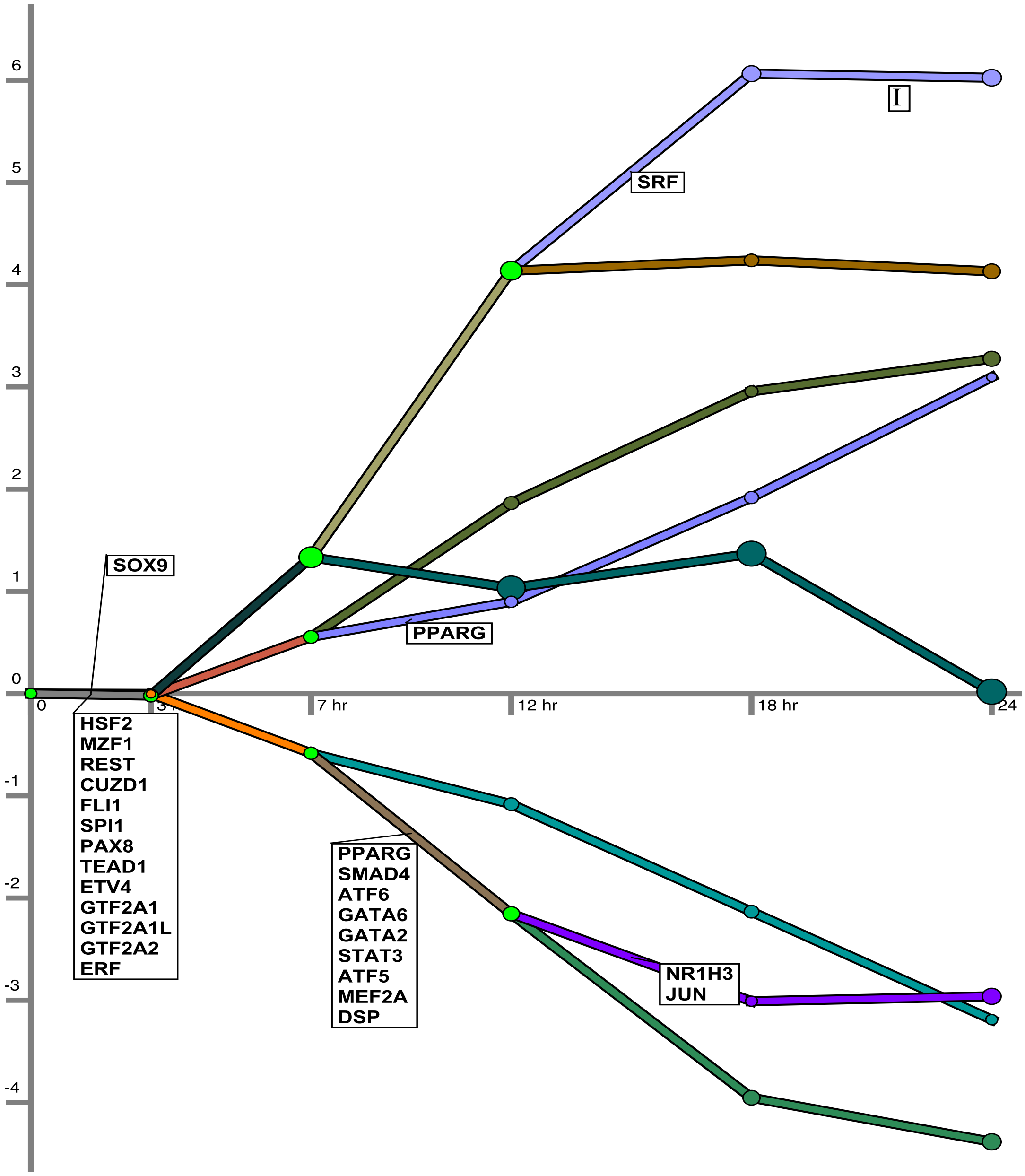

Figure S2. The H5N1 regulatory network is presented. The paths represent the different gene sets which are coexpressed. The TF lists are the TFs predicted to regulate the path that they are connected to.

Strain-specific protein list

H1N1	H3N2	H5N1
CREB1	PCNA	CASP8
PARP1	COPS5	ERBB3
XRCC6	IRF7	COMMD1
POLR2A	SMAD1	PSMA7
CEBPD	ETS1	SPI1
TRIM28	SP3	NUP98
ATM	SMARCB1	HNRNPF
ACTB	TP63	KPNA6
XRCC5	SFPQ	EIF4G1
HSF1	BCR	PCBP1
EEF1A1	SMURF2	DDX39B
ATF4	TRIM27	STAU1
KHDRBS1	ANXA1	IPO5
HNRNPA1	DCTN1	PABPN1
HSP90AB1	CHAF1A	TLR3
DDB1	DVL3	HSPA4
POU2F1	KAT2B	GTF3C3
CRKL	DDX3X	MX1
CRK	RPS3A	GLUL
RPL5	RABGEF1	CCND1
RUVBL2	AIMP2	NQO2
RPL11	SP4	CDKN1B
CDC42	HNRNPH1	TLR8
MCM7	PSMD8	MAPK8
DDX17	MAGEA11	NOMO2
VIM	MLH1	CDKN1A
EWSR1	GSK3B	SIRT1
RPS7	NCOR1	RUNX2
	RBM14	FOS
	PIN1	IVNS1ABP
	NMI	SNAPC4
		ERBB2
		NCOA3
		TRAF6
		TP73
		CASP3
		PRKDC
		CDK1

Table S1. Strain-specific protein list

RNAi screen hits

We compiled primary hits from three genome-wide [2–4] and two targeted [5,6] RNAi screens. The studies varied in the type of human cells, viral strain, functional readout, and other experimental conditions as reviewed in [7]. Brass et al. [2] identified host genes required for viral replication by quantifying the surface expression of the viral hemagglutinin protein. Karlas et al. [3] measured both viral nucleoprotein levels and released viral particles to probe different stages of the viral replication cycle. König et al. [4] detected host factors that inhibit replication efficiency using a modified H1N1 virus that contained a luciferase reporter in place of the hemagglutinin gene. Shapira et al. [5] first identified human proteins that directly interact with viral proteins and their neighbors in the human PPI network as well as human genes that are differentially expressed following viral infection or related treatments. They expanded this set of candidate genes to include related pathway members, and performed three assays to quantify the impact on viral production and interferon beta levels in response to viral RNA transfection or infection by a mutant H1N1 strain. As in [7], we considered only the primary hits that inhibit viral replication upon knockdown. Bortz et al. [6] focused specifically on human genes that modulate viral polymerase activity, screening only a targeted list of human proteins known to interact with H1N1 viral polymerase, nucleoprotein, or ribonucleoprotein that was further refined based on interaction network properties and molecular function. The host genes' impact on viral polymerase activity was separately assessed in two screens: one that transfected human cells with viral polymerase alone and another that infected cells with the complete influenza virus. We included genes that significantly affected polymerase activity in either screen as RNAi hits and collected strain-specific hits for the independent H1N1 and H5N1 screens.

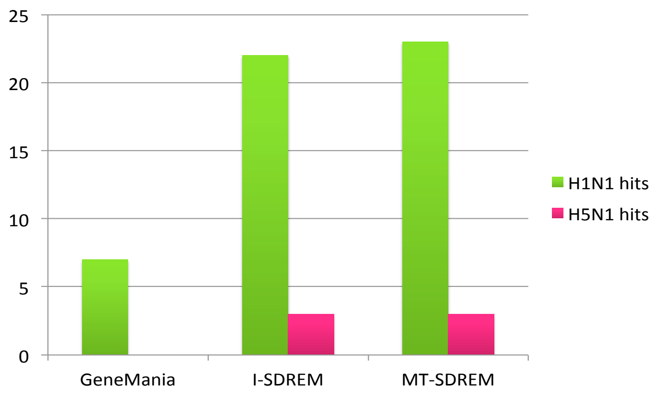

Figure S3. Screen hits overlap for top 100 ranked genes

In Figure S3, we present RNAi screen hits comparison between GeneMania, MT-SDREM, and, I-SDREM. GeneMania outputs a list of genes predicted to be associated with a set of seed genes. We again gave the list of source proteins as the seed genes. Since GeneMania doesn't include the seed genes in its resultant ranking, we compared the ranking output by MT-SDREM and I-SDREM with the *source proteins removed*, against the results output by GeneMania. The results are in Figure S3. GeneMania gets only 7 RNAi screen hits in its top 100 genes. I-SDREM and MT-SDREM do considerably better getting 21 and 23 genes respectively.

In Table S3, we give the the screen hits overlap for the top 100 genes as ranked by Endeavour for the various configurations we ran Endeavour in.

cutoff	I-SDREM	MT-SDREM
50	21	22
100	35	39
150	48	50
200	58	58
250	66	69
300	75	78
350	81	84
400	89	87
450	94	93
500	101	100

Table S2. RNAi hits overlap for H1N1 for a range of cutoffs

Endeavour configuration	Screen hits overlap
Only source genes as training + BioGrid, HPRD as data source	10
Only source genes as training + all data sources used	18
Source and differentially expressed genes as training + BioGrid, HPRD as data sources	15
Source and differentially expressed genes as training + all data sources used	19

Table S3. Screen hits overlap for top 100 genes for Endeavour for H1N1. The corresponding overlap for MT-SDREM is 39.

GSEA comparison

We compared the ranking of MT-SDREM to the ranking output by I-SDREM and by GSEA [8].

Method	Enrichment Score
GSEA ranking	0.34
I-SDREM	0.89
MT-SDREM	0.90

Table S4. Enrichment Score with H1N1 screen hits being the gene enrichment set

Stability of results with respect to α

**H1N1 & H3N2 & H5N1**		**H1N1 & H3N2**		**H3N2 & H5N1**		**H1N1 & H5N1**	
**5**	**6**	**5**	**6**	**5**	**6**	**5**	**6**
AR	AR	IRF1	IRF1			HIF1A	HIF1A
BRCA1	BRCA1	IRF3	IRF3			STAT3	STAT3
EP300	EP300	IRF5	IRF5			TP53	ATF2
ESR1	ESR1	CEBPA	FOSL2				
JUN	JUN	STAT1	TFAP2A				
PPARG	PPARG						
RB1	RB1						
SMAD4	SMAD4						
	CEBPA						
	SOX9						
	STAT1						
	TP53						

Table S5. To demonstrate the stability of the results for different values of α, the TF comparison for $\alpha = 5$ and $\alpha = 6$ is presented.

**H1N1 & H3N2 & H5N1**		**H1N1 & H3N2**		**H3N2 & H5N1**		**H1N1 & H5N1**	
**7**	**6**	**7**	**6**	**7**	**6**	**7**	**6**
AR	AR	IRF1	IRF1			HIF1A	HIF1A
BRCA1	BRCA1	IRF3	IRF3			STAT3	STAT3
EP300	EP300	IRF5	IRF5			ATF2	ATF2
ESR1	ESR1	FOSL2	FOSL2			TBP	
JUN	JUN	CEBPB	TFAP2A				
PPARG	PPARG	CEBPA					
RB1	RB1						
SMAD4	SMAD4						
STAT1	STAT1						
TP53	TP53						
	CEBPA						
	SOX9						

Table S6. To demonstrate the stability of the results for different values of α, the TF comparison for $\alpha = 7$ and $\alpha = 6$ is presented.

α	**RNAi hits overlap**
1	37
2	40
3	37
4	37
5	39
6	39
7	38
8	38
9	41

Table S7. To demonstrate the stability of the results for different values of α, the overlap with RNAi hits for the top 100 genes as ranked by MT-SDREM is presented. The next best method – I-SDREM gets only 35 RNAi hits

Comparison to Tensor Clustering

Note that unless otherwise noted, only a subset of the immune categories are presented as the full list is too large to fit in the supplement. The complete table is available on the supporting website.

Complete Network Used

GO Category	MT-SDREM p-value $\leq$	Tensor p-value	GO Category Description
GO:0046651	0.001	NA	lymphocyte proliferation
GO:0043923	0.001	NA	positive regulation by host of viral transcription
GO:0060070	0.001	NA	canonical Wnt receptor signaling pathway
GO:0019083	0.001	NA	viral transcription
GO:0048524	0.001	NA	positive regulation of viral process
GO:0019042	0.001	NA	viral latency
GO:0019048	0.001	NA	modulation by virus of host morphology or physiology
GO:0019058	0.001	NA	viral life cycle
GO:0051607	0.001	NA	defense response to virus
GO:0009615	0.001	NA	response to virus
GO:0046782	0.001	NA	regulation of viral transcription
GO:0031349	0.001	NA	positive regulation of defense response
GO:0002684	0.001	NA	positive regulation of immune system process
GO:0002429	0.001	NA	immune response-activating cell surface receptor signaling pathway
GO:0002694	0.001	NA	regulation of leukocyte activation
GO:0002697	0.001	NA	regulation of immune effector process
GO:0050778	0.001	NA	positive regulation of immune response
GO:0007259	0.001	NA	JAK-STAT cascade
GO:0050852	0.001	NA	T cell receptor signaling pathway

Table S8. The GO comparison for the **all the edges** in the networks between MT-SDREM and Tensor clustering. Only categories with MT-SDREM p-value < 0.001 and Tensor clustering p-value ≥ 0.01 or not present in Tensor clustering are shown. Only select categories are shown. In total, 109 immune-related categories are present and given in Supplementary Table S35 on the Supporting Website.

We could not find any enriched categories for Tensor clustering with p-value ≤ 0.001 that were either not present in MT-SDREM or for which the p-value in MT-SDREM was ≥ 0.01.

Top 20,000 edges

GO Category	MT-SDREM p-value ≤	Tensor p-value	GO Category Description
GO:0002703	0.001	NA	regulation of leukocyte mediated immunity
GO:0002367	0.001	NA	cytokine production involved in immune response
GO:0002819	0.001	NA	regulation of adaptive immune response
GO:0048524	0.001	NA	positive regulation of viral process
GO:0019083	0.001	NA	viral transcription
GO:0050688	0.001	NA	regulation of defense response to virus
GO:0046651	0.001	NA	lymphocyte proliferation
GO:0042098	0.001	NA	T cell proliferation

Table S9. The GO comparison for the **top 20,000 edges** in the networks between MT-SDREM and Tensor clustering. Only categories with MT-SDREM p-value < 0.001 and Tensor clustering p-value ≥ 0.01 or not present in Tensor clustering are shown. Only select categories are shown. The total number of immune-categories are 98 and given in Supplementary Table S36 on the Supporting Website.

GO Category	Tensor clustering p-value ≤	MT-SDREM p-value	GO Category Description
GO:0045071	0.001	0.021	negative regulation of viral genome replication

Table S10. The GO comparison for the **top 20,000 edges** in the networks between MT-SDREM and Tensor clustering. Only categories with Tensor clutsering p-value < 0.001 and MT-SDREM p-value ≥ 0.01 or not present in MT-SDREM are shown. Note that **all** such immune related categories are shown

Top 5,000 edges

GO Category	MT-SDREM p-value ≤	Tensor p-value	GO Category Description
GO:0002684	0.001	NA	positive regulation of immune system process
GO:0045089	0.001	NA	positive regulation of innate immune response
GO:0051059	0.001	NA	NF-kappaB binding
GO:0007259	0.001	NA	JAK-STAT cascade
GO:0042110	0.001	NA	T cell activation
GO:0019083	0.001	NA	viral transcription
GO:0019058	0.001	NA	viral life cycle
GO:0050792	0.001	NA	regulation of viral process
GO:0034142	0.001	NA	toll-like receptor 4 signaling pathway
GO:0043122	0.001	NA	regulation of I-kappaB kinase/NF-kappaB cascade
GO:0032647	0.001	NA	regulation of interferon-alpha production
GO:0032648	0.001	NA	regulation of interferon-beta production

Table S11. The GO comparison for the **top 5,000 edges** in the networks between MT-SDREM and Tensor clustering. Only categories with MT-SDREM p-value < 0.001 and Tensor clustering p-value ≥ 0.01 or not present for Tensor clustering are shown. Only select categories are shown. The total number of such immune-related categories are 71 and given in Supplementary Table S37 on the Supporting Website.

GO Category	Tensor clustering p-value ≤	MT-SDREM p-value	GO Category Description
GO:0071357	0.001	NA	cellular response to type I interferon
GO:0034340	0.001	NA	response to type I interferon
GO:0060337	0.001	NA	type I interferon-mediated signaling pathway

Table S12. The GO comparison for the **top 5,000 edges** in the networks between MT-SDREM and Tensor clustering. Only categories with Tensor clustering p-value < 0.001 and MT-SDREM p-value ≥ 0.01 or not present for MT-SDREM. Note that **all** such immune related categories are shown

Further comparison to oPossum

we have also tested oPossum with the three different DE gene sets (one for each condition). Below we present the condition-specific comparison to oPossum. As highlighted, MT-SDREM finds far more immune response and flu response related TFs compared to oPossum.

**oPossum**	**MT-SDREM**
Foxd3	TP53
Nkx2-5	EP300
HOXA5	BRCA1
NKX3-1	**JUN**
ARID3A	ESR1
Pdx1	AR
SRY	**RB1**
FOXI1	**STAT1**
Prrx2	**CEBPB**
FOXD1	**CEBPA**
Sox5	CREB1
**NFATC2**	**STAT3**
FOXA1	**IRF1**
Gfi	**ATF2**
HLF	HIF1A
ELF5	**SMAD4**
FOXO3	**IRF3**
Nobox	**NFKB1**
SPIB	**FOSL2**
HNF1B	**IRF5**

Table S13. oPossum vs MT-SDREM for H1N1

**oPossum**	**MT-SDREM**
Nkx2-5	EP300
**IRF2**	TP53
SRY	BRCA1
Foxd3	AR
FOXA1	**JUN**
NKX3-1	ESR1
FEV	**SMAD4**
**IRF1**	**SMAD3**
Foxa2	**STAT1**
HOXA5	**RB1**
TBP	MYC
FOXO3	**CEBPA**
**CEBPA**	**PPARG**
ARID3A	**SMAD2**
Sox5	**IRF1**
FOXI1	**IRF3**
FOXD1	**IRF7**
**AP1**	**FOSL2**
Pou5f1	**SMAD1**
SPI1	ETS1

Table S14. oPossum vs MT-SDREM for H3N2

**oPossum**	**MT-SDREM**
SP1	EP300
Klf4	ESR1
MZF1_5-13	**RB1**
REST	**JUN**
HLF	TP53
Sox5	BRCA1
MZF1_1-4	AR
HNF1B	**SMAD4**
**IRF1**	**STAT3**
Ar	**PPARG**
Hand1::Tcfe2a	**CEBPA**
EBF1	**STAT1**
Nkx2-5	**ATF2**
Pdx1	**RELA**
Nr2e3	HIF1A
Spz1	SOX9
SRF	SPI1
RUNX1	**CEBPB**
TEAD1	**SMAD3**
Arnt::Ahr	**NR3C1**

Table S15. oPossum vs MT-SDREM for H5N1

Further GO comparison to Differentially Expressed (DE) genes

Below we present the GO comparison to the DE genes list for the condition-specific list as well as a comparison based on enrichment using the top 1000 genes instead of just 500.

Top 500 genes

H1N1 GO comparison

GO Category	MT-SDREM p-value $\leq$	DE p-value	GO Category Description
GO:0002684	0.001	NA	positive regulation of immune system process
GO:0002429	0.001	NA	immune response-activating cell surface receptor signaling pathway
GO:0050863	0.001	NA	regulation of T cell activation
GO:0050864	0.001	NA	regulation of B cell activation
GO:0019083	0.001	NA	viral transcription
GO:0019042	0.001	NA	viral latency
GO:0019058	0.001	NA	viral life cycle
GO:0016055	0.001	NA	Wnt receptor signaling pathway
GO:0001959	0.001	NA	regulation of cytokine-mediated signaling pathway
GO:0034138	0.001	NA	toll-like receptor 3 signaling pathway
GO:0002696	0.001	NA	positive regulation of leukocyte activation
GO:0060759	0.001	NA	regulation of response to cytokine stimulus

Table S16. GO comparison between the Differentially Expressed (DE) gene list and MT-SDREM gene list for H1N1 for top 500 genes. The enrichment was performed using the FuncAssociate tool [9]. Only categories with MT-SDREM adjusted p-value of ≤ 0.001 and DE genes p-value of ≥ 0.01 are presented. If a p-value for DE genes is NA, that means that that category was not enriched for in the DE genes list. Only **select** immune response related categories are presented. The full list of the 113 immune-reltaed categories is available in Supplementary Table S38 on the Supporting Website.

GO Category	DE p-value $\leq$	MT-SDREM p-value	GO Category Description
GO:0045071	0.001	NA	negative regulation of viral genome replication
GO:0045069	0.001	0.037	regulation of viral genome replication
GO:0048525	0.001	NA	negative regulation of viral process

Table S17. GO comparison between the DE gene list and the MT-SDREM for H1N1 for the top 500 genes. The enrichment was performed using the FuncAssociate tool [9]. Only categories with DE genes adjusted p-value of ≤ 0.001 and MT-SDREM genes p-value of ≥ 0.01 are presented. If a p-value for MT-SDREM is NA, that means that that category was not enriched for in the MT-SDREM list. **All** immune response related categories are presented.

H3N2 GO comparison

GO Category	MT-SDREM p-value ≤	DE p-value	GO Category Description
GO:0043923	0.001	NA	positive regulation by host of viral transcription
GO:0001959	0.001	NA	regulation of cytokine-mediated signaling pathway
GO:0034134	0.001	NA	toll-like receptor 2 signaling pathway
GO:0042113	0.001	NA	B cell activation
GO:0050852	0.001	NA	T cell receptor signaling pathway
GO:0002429	0.001	NA	immune response-activating cell surface receptor signaling pathway
GO:0045089	0.001	0.034	positive regulation of innate immune response

Table S18. GO comparison between the Differentially Expressed (DE) gene list and MT-SDREM gene list for H3N2 for top 500 genes. The enrichment was performed using the FuncAssociate tool [9]. Only categories with MT-SDREM adjusted p-value of ≤ 0.001 and DE genes p-value of ≥ 0.01 are presented. If a p-value for DE genes is NA, that means that that category was not enriched for in the DE genes list. Only **select** immune response related categories are presented. The full list of the 86 immune-related categories is available in Supplementary Table S39 on the Supporting Website.

GO Category	DE p-value ≤	MT-SDREM p-value	GO Category Description
GO:0045071	0.001	NA	negative regulation of viral genome replication
GO:0045069	0.001	0.01	regulation of viral genome replication
GO:0035456	0.001	NA	response to interferon-beta
GO:0048525	0.001	NA	negative regulation of viral process

Table S19. GO comparison between the DE gene list and the MT-SDREM for H3N2 for the top 500 genes. The enrichment was performed using the FuncAssociate tool [9]. Only categories with DE genes adjusted p-value of ≤ 0.001 and MT-SDREM genes p-value of ≥ 0.01 are presented. If a p-value for MT-SDREM is NA, that means that that category was not enriched for in the MT-SDREM list. **All** immune response related categories are presented.

H5N1 GO comparison

GO Category	MT-SDREM p-value ≤	DE p-value	GO Category Description
GO:0002366	0.001	NA	leukocyte activation involved in immune response
GO:0002218	0.001	NA	activation of innate immune response
GO:0043921	0.001	NA	modulation by host of viral transcription
GO:0043923	0.001	NA	positive regulation by host of viral transcription
GO:0048524	0.001	NA	positive regulation of viral process
GO:0019221	0.001	NA	cytokine-mediated signaling pathway
GO:0032727	0.001	NA	positive regulation of interferon-alpha production
GO:0070661	0.001	NA	leukocyte proliferation

Table S20. GO comparison between the Differentially Expressed (DE) gene list and MT-SDREM gene list for H5N1 for top 500 genes. The enrichment was performed using the FuncAssociate tool [9]. Only categories with MT-SDREM adjusted p-value of ≤ 0.001 and DE genes p-value of ≥ 0.01 are presented. If a p-value for DE genes is NA, that means that that category was not enriched for in the DE genes list. Only **select** immune response related categories are presented. The full list of the 145 immune-related categories is available in Supplementary Table S40 on the Supporting Website.

No categories were found for H5N1 for which the p-value of DE gene list was ≤ 0.001 and the category was either not present or its p-value for the MT-SDREM list was ≥ 0.01. By contrast, MT-SDREM had 145 immune-related categories with p-value ≤ 0.001 listed in Supplementary Table S40.

Top 1000 genes

Joint list GO comparison

GO Category	MT-SDREM p-value $\leq$	DE p-value	GO Category Description
GO:0002376	0.001	NA	immune system process
GO:0002366	0.001	NA	leukocyte activation involved in immune response
GO:0002218	0.001	NA	activation of innate immune response
GO:0006952	0.001	NA	defense response
GO:0043123	0.001	NA	positive regulation of I-kappaB kinase/NF-kappaB cascade
GO:0032727	0.001	NA	positive regulation of interferon-alpha production
GO:0050792	0.001	NA	regulation of viral process
GO:0019080	0.001	NA	viral genome expression
GO:0019083	0.001	NA	viral transcription

Table S21. GO comparison between the Differentially Expressed (DE) gene list combined using the Kemeny method and MT-SDREM gene list combined using the flow sum method as described in the main manuscript. The enrichment was performed using the FuncAssociate tool [9]. Only categories with MT-SDREM adjusted p-value of ≤ 0.001 and DE genes p-value of ≥ 0.01 are presented. If a p-value for DE genes is NA, that means that that category was not enriched for in the DE genes list. Only **select** immune response related categories are presented. The full list of the 158 immune-related categories is available in Supplementary Table S41 on the Supporting Website.

GO Category	DE p-value $\leq$	MT-SDREM p-value	GO Category Description
GO:0045071	0.001	NA	negative regulation of viral genome replication

Table S22. GO comparison between the Differentially Expressed (DE) gene list combined using the Kemeny method and MT-SDREM gene list combined using the flow sum method as described in the main manuscript. The enrichment was performed using the FuncAssociate tool [9]. Only categories with DE genes adjusted p-value of ≤ 0.001 and MT-SDREM genes p-value of ≥ 0.01 are presented. If a p-value for MT-SDREM is NA, that means that that category was not enriched for in the MT-SDREM list. **All** immune response related categories are presented.

H1N1 GO comparison

GO Category	MT-SDREM p-value ≤	DE p-value	GO Category Description
GO:0002684	0.001	NA	positive regulation of immune system process
GO:0002703	0.001	NA	regulation of leukocyte mediated immunity
GO:0002367	0.001	NA	cytokine production involved in immune response
GO:0002366	0.001	NA	leukocyte activation involved in immune response
GO:0002218	0.001	NA	activation of innate immune response
GO:0007259	0.001	NA	JAK-STAT cascade
GO:0045580	0.001	NA	regulation of T cell differentiation
GO:0042113	0.001	NA	B cell activation
GO:0042110	0.001	NA	T cell activation

Table S23. GO comparison between the Differentially Expressed (DE) gene list and MT-SDREM gene list for H1N1 for top 1000 genes. The enrichment was performed using the FuncAssociate tool [9]. Only categories with MT-SDREM adjusted p-value of ≤ 0.001 and DE genes p-value of ≥ 0.01 are presented. If a p-value for DE genes is NA, that means that that category was not enriched for in the DE genes list. Only **select** immune response related categories are presented. The full list of the 163 immune-related categories is available in Supplementary Table S42 on the Supporting Website.

GO Category	DE p-value ≤	MT-SDREM p-value	GO Category Description
GO:0045071	0.001	NA	negative regulation of viral genome replication

Table S24. GO comparison between the DE gene list and the MT-SDREM for H1N1 for the top 1000 genes. The enrichment was performed using the FuncAssociate tool [9]. Only categories with DE genes adjusted p-value of ≤ 0.001 and MT-SDREM genes p-value of ≥ 0.01 are presented. If a p-value for MT-SDREM is NA, that means that that category was not enriched for in the MT-SDREM list. **All** immune response related categories are presented.

H3N2 GO comparison

GO Category	MT-SDREM p-value ≤	DE p-value	GO Category Description
GO:0002703	0.001	0.029	regulation of leukocyte mediated immunity
GO:0042098	0.001	NA	T cell proliferation
GO:0060070	0.001	NA	canonical Wnt receptor signaling pathway
GO:0043921	0.001	NA	modulation by host of viral transcription
GO:0019080	0.001	NA	viral genome expression
GO:0019083	0.001	NA	viral transcription
GO:1902107	0.001	NA	positive regulation of leukocyte differentiation
GO:0001959	0.001	NA	regulation of cytokine-mediated signaling pathway

Table S25. GO comparison between the Differentially Expressed (DE) gene list and MT-SDREM gene list for H3N2 for top 1000 genes. The enrichment was performed using the FuncAssociate tool [9]. Only categories with MT-SDREM adjusted p-value of ≤ 0.001 and DE genes p-value of ≥ 0.01 are presented. If a p-value for DE genes is NA, that means that that category was not enriched for in the DE genes list. Only **select** immune response related categories are presented. The full list of the 102 immune-related categories is available in Supplementary Table S43 on the Supporting Website.

GO Category	DE p-value ≤	MT-SDREM p-value	GO Category Description
GO:0045071	0.001	NA	negative regulation of viral genome replication
GO:0048525	0.001	0.039	negative regulation of viral process

Table S26. GO comparison between the DE gene list and the MT-SDREM for H3N2 for the top 1000 genes. The enrichment was performed using the FuncAssociate tool [9]. Only categories with DE genes adjusted p-value of ≤ 0.001 and MT-SDREM genes p-value of ≥ 0.01 are presented. If a p-value for MT-SDREM is NA, that means that that category was not enriched for in the MT-SDREM list. **All** immune response related categories are presented.

H5N1 GO comparison

GO Category	MT-SDREM p-value ≤	DE p-value	GO Category Description
GO:0002218	0.001	NA	activation of innate immune response
GO:0045089	0.001	NA	positive regulation of innate immune response
GO:0043921	0.001	NA	modulation by host of viral transcription
GO:0016032	0.001	NA	viral process
GO:0050852	0.001	NA	T cell receptor signaling pathway
GO:0050853	0.001	NA	B cell receptor signaling pathway

Table S27. GO comparison between the Differentially Expressed (DE) gene list and MT-SDREM gene list for H5N1 for top 1000 genes. The enrichment was performed using the FuncAssociate tool [9]. Only categories with MT-SDREM adjusted p-value of ≤ 0.001 and DE genes p-value of ≥ 0.01 are presented. If a p-value for DE genes is NA, that means that that category was not enriched for in the DE genes list. Only **select** immune response related categories are presented. The full list of the 188 immune-related categories is available in Supplementary Table S44 on the Supporting Website.

No GO enrichment at all was found for the top 1000 DE genes for H5N1.

Strain-specific statistics

Category	I-SDREM			MT-SDREM		
# of common proteins	39			39		
	H1N1	H3N2	H5N1	H1N1	H3N2	H5N1
# of strain-specific proteins	28	31	38	29	32	34
# of RNAi hits for strain-specific proteins	8		1	11		1

Table S28. The number of strain-specific proteins and their overlap with RNAi screen hits is given.

GO comparison with I-SDREM

Below we present the condition-specific GO comparison with I-SDREM for the top 500 genes. The FuncAssociate tool was used for the GO comparison. While the I-SDREM list gives slightly better GO categories compared to the MT-SDREM list, we'd like to note that the opposite can be true when the DAVID tool [10, 11] is used for comparison. There, the MT-SDREM list gives slightly better categories. Thus we conclude that the GO differences between the two tools are not significant.

H1N1 comparison

MT-SDREM finds 163 immune related categories while I-SDREM finds 182 such categories with FuncAssociate. Some of those categories are presented below – the full list is on the Supporting website. (By contrast, when using DAVID, MT-SDREM finds 296 immune related categories while I-SDREM finds only 282).

GO Category	MT-SDREM p-value ≤	GO Category Description
GO:0033256	0.001	I-kappaB/NF-kappaB complex
GO:0002326	0.013	B cell lineage commitment
GO:0045586	0.037	regulation of gamma-delta T cell differentiation
GO:0045588	0.037	positive regulation of gamma-delta T cell differentiation
GO:0070411	0.001	I-SMAD binding
GO:0042090	0.007	interleukin-12 biosynthetic process
GO:0045075	0.007	regulation of interleukin-12 biosynthetic process
GO:0019042	0.001	viral latency
GO:0032727	0.001	positive regulation of interferon-alpha production
GO:0032607	0.001	interferon-alpha production

Table S29. Some immune response related GO categories for MT-SDREM H1N1 genes

GO Category	I-SDREM p-value ≤	GO Category Description
GO:0045351	0.001	type I interferon biosynthetic process
GO:0019043	0.018	establishment of viral latency
GO:0035666	0.001	TRIF-dependent toll-like receptor signaling pathway
GO:0002902	0.001	regulation of B cell apoptotic process
GO:0060334	0.001	regulation of interferon-gamma-mediated signaling pathway
GO:0043923	0.028	positive regulation by host of viral transcription
GO:0002756	0.001	MyD88-independent toll-like receptor signaling pathway
GO:0034138	0.001	toll-like receptor 3 signaling pathway
GO:0060330	0.001	regulation of response to interferon-gamma
GO:0034146	0.001	toll-like receptor 5 signaling pathway

Table S30. Some immune response related GO categories for I-SDREM H1N1 genes

H3N2 comparison

MT-SDREM finds 164 immune related categories while I-SDREM finds 187 such categories with FuncAssociate. Some of those categories are presented below – the full list is on the Supporting website. (By contrast, when using DAVID, MT-SDREM finds 348 immune related categories while I-SDREM finds 354).

GO Category	MT-SDREM p-value ≤	GO Category Description
GO:0032727	0.001	positive regulation of interferon-alpha production
GO:0043923	0.001	positive regulation by host of viral transcription
GO:0032607	0.001	interferon-alpha production
GO:0032647	0.001	regulation of interferon-alpha production
GO:0045351	0.001	type I interferon biosynthetic process
GO:0035666	0.001	TRIF-dependent toll-like receptor signaling pathway
GO:0070412	0.001	R-SMAD binding
GO:0002756	0.001	MyD88-independent toll-like receptor signaling pathway
GO:0032728	0.001	positive regulation of interferon-beta production
GO:0007183	0.017	SMAD protein complex assembly

Table S31. Some immune response related GO categories for MT-SDREM H3N2 genes

GO Category	I-SDREM p-value ≤	GO Category Description
GO:0034146	0.001	toll-like receptor 5 signaling pathway
GO:0032728	0.001	positive regulation of interferon-beta production
GO:0034166	0.001	toll-like receptor 10 signaling pathway
GO:0032481	0.001	positive regulation of type I interferon production
GO:0002724	0.003	regulation of T cell cytokine production
GO:0034162	0.001	toll-like receptor 9 signaling pathway
GO:0070412	0.001	R-SMAD binding
GO:0034142	0.001	toll-like receptor 4 signaling pathway
GO:0060395	0.001	SMAD protein signal transduction
GO:1901222	0.001	regulation of NIK/NF-kappaB cascade

Table S32. Some immune response related GO categories for I-SDREM H3N2 genes

H5N1 comparison

MT-SDREM finds 189 immune related categories while I-SDREM finds 174 such categories with FuncAssociate. Some of those categories are presented below – the full list is on the Supporting website. (By contrast, when using DAVID, MT-SDREM finds 370 immune related categories while I-SDREM finds 374).

GO Category	MT-SDREM p-value ≤	GO Category Description
GO:0032606	0.001	type I interferon production
GO:0060397	0.001	JAK-STAT cascade involved in growth hormone signaling pathway
GO:0001961	0.002	positive regulation of cytokine-mediated signaling pathway
GO:0032728	0.002	positive regulation of interferon-beta production
GO:0051059	0.002	NF-kappaB binding
GO:0032479	0.001	regulation of type I interferon production
GO:0060760	0.004	positive regulation of response to cytokine stimulus
GO:0045089	0.001	positive regulation of innate immune response
GO:0051092	0.001	positive regulation of NF-kappaB transcription factor activity
GO:0032088	0.001	negative regulation of NF-kappaB transcription factor activity

Table S33. Some immune response related GO categories for MT-SDREM H5N1 genes

GO Category	I-SDREM p-value ≤	GO Category Description
GO:0002218	0.001	activation of innate immune response
GO:0019048	0.001	modulation by virus of host morphology or physiology
GO:0032480	0.001	negative regulation of type I interferon production
GO:0032608	0.001	interferon-beta production
GO:0051092	0.001	positive regulation of NF-kappaB transcription factor activity
GO:0045089	0.001	positive regulation of innate immune response
GO:0060338	0.001	regulation of type I interferon-mediated signaling pathway
GO:0045088	0.001	regulation of innate immune response
GO:0046332	0.001	SMAD binding
GO:0032088	0.001	negative regulation of NF-kappaB transcription factor activity

Table S34. Some immune response related GO categories for I-SDREM H5N1 genes

References

1. Gitter A, Klein-Seetharaman J, Gupta A, Bar-Joseph Z (2011) Discovering pathways by orienting edges in protein interaction networks. Nucleic acids research 39: e22–e22.

2. Brass AL, Huang I, Benita Y, John SP, Krishnan MN, et al. (2009) The ifitm proteins mediate cellular resistance to influenza a h1n1 virus, west nile virus, and dengue virus. Cell 139: 1243–1254.

3. Karlas A, Machuy N, Shin Y, Pleissner KP, Artarini A, et al. (2010) Genome-wide rnai screen identifies human host factors crucial for influenza virus replication. Nature 463: 818–822.

4. König R, Stertz S, Zhou Y, Inoue A, Hoffmann HH, et al. (2009) Human host factors required for influenza virus replication. Nature 463: 813–817.

5. Shapira SD, Gat-Viks I, Shum BO, Dricot A, de Grace MM, et al. (2009) A physical and regulatory map of host-influenza interactions reveals pathways in h1n1 infection. Cell 139: 1255–1267.

6. Bortz E, Westera L, Maamary J, Steel J, Albrecht RA, et al. (2011) Host-and strain-specific regulation of influenza virus polymerase activity by interacting cellular proteins. MBio 2: e00151–11.

7. Stertz S, Shaw ML (2011) Uncovering the global host cell requirements for influenza virus replication via rnai screening. Microbes and Infection 13: 516–525.

8. Subramanian A, Tamayo P, Mootha VK, Mukherjee S, Ebert BL, et al. (2005) Gene set enrichment analysis: a knowledge-based approach for interpreting genome-wide expression profiles. Proceedings of the National Academy of Sciences of the United States of America 102: 15545–15550.

9. Berriz GF, King OD, Bryant B, Sander C, Roth FP (2003) Characterizing gene sets with funcassociate. Bioinformatics 19: 2502–2504.

10. Da Wei Huang BTS, Lempicki RA (2008) Systematic and integrative analysis of large gene lists using david bioinformatics resources. Nature protocols 4: 44–57.

11. Huang DW, Sherman BT, Lempicki RA (2009) Bioinformatics enrichment tools: paths toward the comprehensive functional analysis of large gene lists. Nucleic acids research 37: 1–13.
